# Positive regulation of a LuxR family protein, MilO, in mildiomycin biosynthesis

**DOI:** 10.1128/aem.01654-24

**Published:** 2024-12-23

**Authors:** Zhiyu Li, Yuli Wang, Chen Lin, Yu Wen, Zixin Deng, Ming Jiang, Xinyi He

**Affiliations:** 1 State Key Laboratory of Microbial Metabolism, Joint International Research Laboratory of Metabolic & Developmental Sciences, School of Life Sciences & Biotechnology, Shanghai Jiao Tong University 553742 , Shanghai, People's Republic of China; Danmarks Tekniske Universitet The Novo Nordisk Foundation Center for Biosustainability, Kgs. Lyngby, Denmark

**Keywords:** MilO, LuxR regulator, mildiomycin, gene overexpression, yield improvement, shunt product, *Streptomyces avermitilis*

## Abstract

Mildiomycin is a representative peptidyl nucleoside antibiotic and was first isolated from *Streptoverticillium rimofaciens*, which has been used as an important biological agent to control powdery mildew in plants. Despite its importance, the biosynthetic pathways and regulatory mechanisms remain to be fully elucidated. In this study, we identified MilO as a positive pathway-specific regulator of mildiomycin biosynthesis in the heterologous host *Streptomyces avermitilis*. Gene disruption of *milO* resulted in almost loss of mildiomycin production, and it was restored to the level comparable to that in the wild-type strain in complemented strain. Overexpression of *milO* using host native promoter *rpsJ*p, engineered promotor *SP44,* and *kasO*p* led to a 50%, 6.5-fold, and 9.2-fold increase in mildiomycin production compared with the wild-type strain, respectively. Quantitative real-time PCR and electrophoretic mobility shift assay (EMSA) experiments revealed that MilO directly enhances the transcription of the *milA* gene by 20 folds after 48 h fermentation and indirectly regulates the transcription levels of other genes from *milB* to *milM*. Using DNase I footprinting assays, *milO* was revealed to bind to a 44 bp DNA sequence of the *milA* promoter region. The binding region consists of three imperfect direct repeats of TGTC(N)_3_CGGT separated by two-nucleotide spacers and each repeat is important to efficient binding to MilO. In addition, we identified two related compounds by overexpressing *milO* in a structural gene *milN*-deficient mutant. Taken together, this study indicates that pathway-specific regulator MilO is essential for mildiomycin biosynthesis and provides an effective strategy to improve the production of mildiomycin and its intermediates.

**IMPORTANCE:**

As an important biological agent to control powdery mildew on plants, mildiomycin has been commercialized and used in various plants. However, its regulatory mechanisms and biosynthetic pathways remain unknown. This study provides new insights into the regulation of mildiomycin biosynthesis through MilO, a LuxR family protein that modulates mildiomycin production by directly enhancing the transcription of *milA*. The yield of mildiomycin was significantly improved by overexpressing *milO* in a heterologous host. In addition, the positive regulatory effect of *milO* helped to discover two related compounds, which provide important clues for the timing of uploading of two amino acid side chains during mildiomycin biosynthesis for the first time. In brief, our findings on transcriptional regulation of mildiomycin biosynthesis by *milO* will be valuable to further increase the yield of mildiomycin and explore its biosynthetic pathways.

## INTRODUCTION

**Fig 1:**
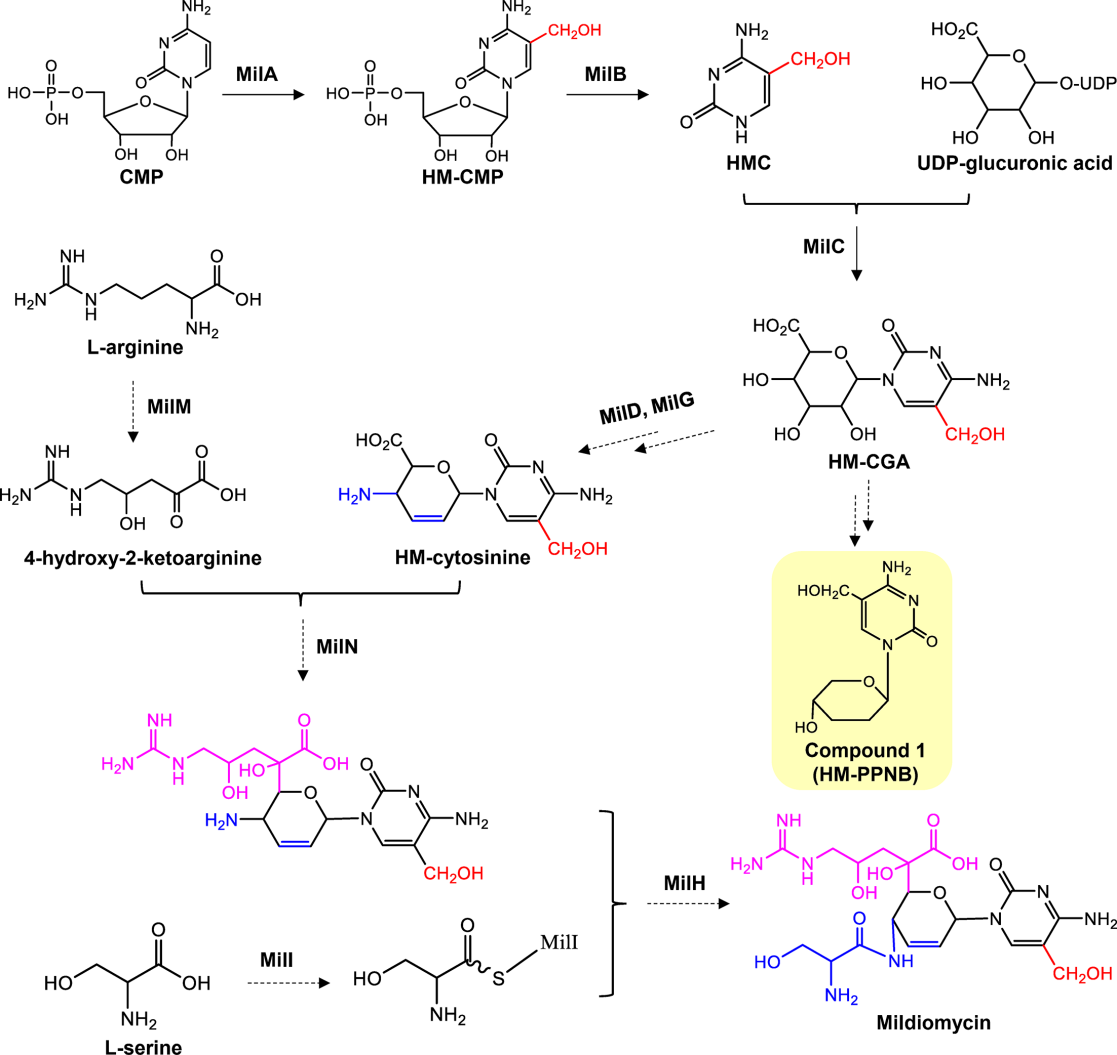
Proposed biosynthetic pathway of mildiomycin. CMP, cytidine 5′-monophosphate. HM-CMP, 5-hydroxymethyl CMP. HMC, 5-hydroxymethylcytosine. HM-CGA, 5-hydroxymethyl cytosylglucuronic acid. MilA, CMP 5-hydroxymethylase. MilB, nucleotide hydrolase. MilC, cytosylglucuronic acid/hydroxymethyl cytosylglucuronic acid synthase. MilD, putative degT/dnrJ/eryC1/strS aminotransferase. MilG, putative cytosylglucuronate decarboxylase. MilM, putative pyridoxal phosphate–dependent arginine oxidases. MilN, putative dihydrodipicolinate synthetase. MilI, putative acyl carrier protein. MilH, putative tRNA-dependent aminoacyltransferase. The solid arrows represent reactions that have been elucidated, and the dashed arrows indicate proposed reactions. The changing groups are indicated in different colors. The shunt product compound **1** is highlighted in a yellow rectangle.

Peptidyl nucleoside antibiotics are a large family of microbial natural products composed of a nucleobase, a core saccharide, and appended amino acid(s) (1). Because nucleosides and nucleotides play essential roles in most of the fundamental cellular metabolism, peptidyl nucleoside antibiotics exhibit a broad spectrum of biological activities, such as antibacterial, antifungal, antiviral, insecticidal, immunostimulative, immunosuppressive, and antitumor activities (2). Mildiomycin (MIL) is a representative peptidyl nucleoside antibiotic and was first isolated from *Streptoverticillium rimofaciens* in 1978 (3). It features a glucuronic acid-derived C2′, C3′-didehydrated pyranose ring coupled to hydroxymethyl cytosine as the nucleoside moiety, and contains a modified arginine side chain and a serine residue as the peptidyl moiety (Fig. 1) (4). As MIL shows strong activity against powdery mildew on various plants by inhibiting fungal protein biosynthesis and remarkably low toxicity in mammals and fishes, it has been produced and sold commercially in Japan as a fungicide for agricultural and horticulture use (5). However, due to the low yield of its producing strains and the high cost of production from the wild-type *Streptoverticillium rimofaciens*, the application scope of MIL is limited.

The promising biological activity and structural features of MIL attracted interest in its biosynthetic studies. Its biosynthetic gene cluster (BGC) from *Streptoverticillium rimofaciens* has been cloned and successfully expressed in *Streptomyces lividans* although with a relatively low yield (6). The BGC of MIL is about 20 kb consisting of 17 genes (7). Previous biosynthetic studies of MIL have been mainly limited to the formation of 5-hydroxymethylcytosine (HMC) and various strategies to increase the production of MIL (6–12). *In vivo* genetic deletion experiments demonstrated that all the genes except *milL* are essential to MIL biosynthesis (6). However, most genetic disruption mutants could not accumulate evident intermediates associated with MIL biosynthesis, which is partially related to the low yield of MIL (6). No accumulation of related metabolite in a lot of genetic disruption mutants impeded further biosynthetic studies of MIL. Up to date, only MilA, MilB, and MilC have been characterized both *in vivo* and *in vitro*. MilA converts cytidine 5′-monophosphate (CMP) into 5-hydroxymethyl CMP (HM-CMP), MilB hydrolyzes HM-CMP (or CMP) into hydroxymethyl cytosine (or cytosine), which are coupled with uridine diphosphateglucuronic acid (UDP-glucuronic acid) into 5-hydroxymethyl cytosyl-glucuronic acid (HM-CGA) or CGA by MilC, respectively (6–9). The following steps in MIL biosynthesis have not been fully elucidated and the functions of the putatively involved proteins have only been proposed through genetic and bioinformatics studies (Fig. 1; Table S1). MilD and MilG are predicated as transaminase and cytosylglucuronate decarboxylase, respectively, and speculated to convert the C4′ hydroxyl group to an amino group and participate in the formation of the C2′, C3′ double bond of the pyranose ring (Fig. 1) (6). MilM and MilN were hypothesized to be responsible for the modification and loading of the arginine side chain. MilH and MilI were proposed to activate and load the serine side chain (Fig. 1) (6). Thus, it is important to increase the yield of MIL for both its biosynthetic studies and industrial applications.

Many approaches have been developed to improve the production of natural products in *Streptomyces* such as mutagenesis, fermentation optimization, and genetic engineering (13, 14). Mutation breeding, protoplast fusion, and process condition optimization have been applied to enhance the yield of MIL (10–12, 15). However, these methodologies are time-consuming and labor-intensive due to the difficulty in the operation and the degeneration of the original strain. In comparison, with the genetic information available rational genetic engineering provides a more efficient strategy for titer improvement. The commonly used rational metabolic engineering approaches include enhancing the supply of biosynthetic precursors or cofactors, overexpressing efflux or resistance genes, and manipulating the regulation of biosynthetic pathways (13). The biosynthesis of natural products from *Streptomyces* is usually strictly controlled by different regulators (16). Deletion of cluster-situated negative regulators and constitutive expression of activators are technically practical, easy, and usually effective approaches to increase the production of natural products (17, 18). However, transcription engineering mainly relies on the understanding of the regulatory system of the BGC.

In this study, we disclose the mechanism of the biosynthetic pathway regulation in MIL biosynthesis. A LuxR-like family regulator encoded by *milO* was characterized as a pathway-specific regulator in MIL biosynthesis through targeted gene disruption and functional complementation. Moreover, overexpression of *milO* under different promoters significantly increases the production of MIL in heterologous hosts. Quantitative real-time PCR (qPCR) demonstrated that the majority of the *mil* genes are positively regulated by MilO. We found that there was only one MilO binding site, which is located 142 bp upstream of the transcription start site of *milA*. In addition, the binding sequence of MilO, a 44 bp sequence including three 11 bp imperfect direct repeats, has been identified in the promoter region of *milA* by DNase I footprinting experiments. These results will help to further increase the yield and fully understand the biosynthesis of MIL in the future. Overexpression of *milO* in *milN*-deficient mutant results in the accumulation of two compounds, enabling us to determine the uploading timing of two amino acids-derived side chains.

## MATERIALS AND METHODS

### Bacterial strains and growth conditions

*Streptomyces avermitilis* NRRL 8165 in this study was used as the host for heterologous expression. *S. avermitilis* and its derivatives were plated on MS medium (2.0% soya flour, 2.0% mannitol, and 2.0% agar, pH7.2–7.4) for sporulation and cultured in TSBY liquid medium (3.0% tryptic soy broth, 10.3% sucrose, and 0.5% yeast extract) for mycelium harvest. *Escherichia coli* was cultured in LB medium (1% trypton, 1% NaCl, 0.5% yeast extract) supplied with appropriate antibiotics (kanamycin, 50 µg/mL; chloramphenicol, 25 µg/mL; spectinomycin, 50 µg/mL; ampicillin, 50 µg/mL) as required at 37°C. All strains and plasmids used in this study are listed in Table S2.

### Gene deletion, complementation, and overexpression

The *milO* in-frame deletion was carried out on cosmid 14A6 which contains the complete *mil* BGC by the λ-RED mediated PCR targeting method (19). An ampicillin resistance cassette (*bla* gene) was amplified with primers 14A6-KO-F/R (Table S3) including *Pac*I restriction site (5′-TTAATTAA-3′) and a 39-nt homologous extension homologous to the upstream and downstream sequence of *milO* gene, respectively. The cassette was introduced into *E. coli* BW25113/pIJ790/14A6 to substitute *milO* on cosmid 14A6. The correct recombinant cosmid was digested with *Pac*I and subsequently self-ligated with T4 ligase to obtain cosmid 14A6Δ*milO*. Then the mutant cosmid was introduced into *E. coli* ET12567/pUZ8002 and then transferred into *S. avermitilis* by conjugation.

Gene cloning was performed based on the integrative vector pPM927 to address the purposes of gene complementation and overexpression. The gene *milO* was amplified from cosmid 14A6 and placed downstream of the promoters *kasO*p*, *SP44,* and *rpsJ*p by overlapping PCR to obtain fragments *kasO*p*-*milO*, *SP44-milO,* and *rpsJ*p-*milO*, respectively (20, 21). These three fragments were purified and inserted into *EcoR*I-digested pPM927 to generate pPM927-1, pPM927-2, and pPM927-3. The first of them was introduced into the *milO*-deletion strain by conjugative transfer to complete complementation. All of them were introduced into the wild-type (WT) strain *S. avermitilis*::14A6 individually to achieve overexpression. Mutants associated with *milN* were constructed in the same way as that for *milO*-derived strains.

### Fermentation and high-performance liquid chromatography analysis

*S. avermitilis* NRRL 8165 and its derivatives were grown on MS medium at 30°C for 4 days and then inoculated into 250 mL flasks containing 30 mL seed medium (2.0% glucose, 2.0% soya flour, 0.5% yeast extract, 0.5% MgSO_4_·7H_2_O, 0.3% CaCO_3_, and 0.1% (NH_4_)_2_SO_4_, pH7.2–7.6) for another 48 h cultivation at 30°C. Next, 3 mL aforesaid seed culture was inoculated into 250 mL flasks containing 30 mL fermentation medium (8.0% glucose, 3.0% soya flour, 0.6% MgSO_4_·7H_2_O, 1.0% CaCO_3_, and 0.1% (NH_4_)_2_SO_4_, 0.06% FeSO_4_·7H_2_O, 0.04% K_2_HPO_4,_ and 1% N-N-dimethyl acetamide, pH7.2–7.6) (7). Then the fermentation was conducted at 30°C and 200 rpm for 7 days. The pH of the end fermentation broth was adjusted to 3 with oxalic acid to remove impurities, then the supernatant was collected after centrifugation, and transferred into a 50 mL tube containing 5 g cation exchange resin D113 (Chinaresin, China) that was pre-activated with hydrochloric acid (1 M), followed by shaking at 30°C and 220 rpm for 1 h. After that, the supernatant was removed and the resin was rinsed with 100 mL of deionized water to remove the residual sample solution. Subsequently, the resin was desorbed by 10 mL 2% NH_3_·H_2_O. The eluate was concentrated by rotary evaporation and then analyzed by high-performance liquid chromatography (HPLC). HPLC analysis was performed using a TC-C18 column (5 µm, 250 × 4.6 mm; Agilent) on an Agilent 1260 Infinity series system at a flow rate of 0.8 mL min^−1^ at room temperature with the following parameters: solvent A, 10 mM trichloroacetic acid; solvent B, MeOH; gradient, 5% B for 65 min; detection by UV absorbance at 279 nm. The HPLC-purified compounds were further analyzed through Agilent 6546 accurate-mass quadrupole time-of-flight mass spectrometer (Q-TOF-MS) at a flow rate of 0.4 mL min^−1^ with the parameters as follows: TC-C18 column (5 µm, 250 × 4.6 mm; Agilent) at room temperature; solvent A, 0.1% formic acid; solvent B, MeOH; gradient, 10% B for the whole process. The Q-TOF mass spectrometer was equipped with a Dual AJS ESI source operating in positive ionization mode, with a scan range from 100 to 3,200 m/z. ESI ion source parameters were set as follows: ion source drying temperature, 320°C; nitrogen flow, 8 L/min; and capillary voltage, 3,500 V (positive ion mode). The fragmentor voltage was 45 V.

### Biomass measurement of *S*. *avermitilis*-derived strains

By virtue of the insoluble particles in the fermentation medium of MIL, the cell growth of *S. avermitilis*-derived strains was quantified by a simplified diphenylamine colorimetric method (22). 1 mL fermentation broth was collected every 24 h for seven consecutive days and centrifuged at 12,000 *g* for 10 min. The supernatant was retained for MIL yield quantification through HPLC, and the precipitate was washed three times with Milli-Q water for DNA concentration assay. The washed precipitated cells were resuspended and incubated in the diphenylamine reaction buffer (1.5 g diphenylamine, 1.5 mL concentrated sulfuric acid, 1.0 mL 1.6% aqueous acetaldehyde, making up to 100 mL with glacial acetic acid) for 1 h at 60°C, followed by centrifugation for 2 min at 12,000 *g* to retain the supernatant for the measurements of OD_595_. Three biological replicates were used.

### Transcriptional analysis by quantitative real-time PCR

Total RNA was extracted after 48 h and 96 h fermentation by MolPure Bacterial RNA Kit (YEASEN, China), and the RNA concentration was determined through a Nano Drop 2000 spectrophotometer. The cDNA was synthesized using DNase I-treated RNA (1 µg) by Hifair III 1st Strand cDNA Synthesis SuperMix for qPCR Kit (YEASEN, China). Then qPCR of several representative genes and reference gene *hrdB* was performed using specific primers (Table S3) on the Applied Biosystems 7500 Real-Time system (Applied Biosystems, USA) with Hieff qPCR SYBR Green Master Mix (YEASEN, China) to measure the relative transcriptional levels using the 2^−ΔΔCT^ method (23). Three biological replicates were used for each qPCR analysis.

### Determination of transcription units in the BGC of MIL

Fermentation samples of the WT strain were collected at 24 h for the subsequent RNA extraction and reverse transcription as described above. The transcription units in the BGC of MIL were certified by PCR using primers shown in Table S3 with cDNA as the template. DNase I-treated RNA and genomic DNA were used as negative and positive controls, respectively, to ensure that cDNA had no residual genomic DNA and the primers were of high amplification efficiency and specificity. All PCR products were examined by electrophoresis on a 0.8% agarose gel.

### Expression and purification of MilO

DNA fragments encoding MilO were amplified by PCR and cloned into the vector pSJ8 with N-terminal MBP and 8 × His tags (Table S2). The protein expression vector pSJ8-MBP-His_8_-MilO and the control vector pSJ8-MBP-His_8_ were transformed into *E. coli* BL21(DE3). Next, 10 mL overnight culture from a single colony was inoculated into 1 L LB medium supplied with 50 µg/mL ampicillin. After incubation at 37°C to an OD_600_ of 0.6–0.8 and cooling to room temperature, the culture was induced by 0.2 mM isopropyl-D-1-thiogalactopyranoside (IPTG), followed by incubation for another 20 h at 16°C. Next, the cells were centrifuged, resuspended in Ni^2+^ column binding buffer (20 mM Tris, 300 mM NaCl, 20 mM imidazole, pH 8.0), and lysed by high-pressure homogenizer at 4^o^C. After centrifugation (16,000 × *g* for 30 min at 4°C), the supernatant was applied to a Ni-NTA column (GE, USA) pre-equilibrated with binding buffer, and the proteins were eluted with 10 mL Ni^2+^ column elution buffer (20 mM Tris-HCl, 300 mM imidazole and 300 mM NaCl, pH 8.0) after washing. The eluted protein was further purified on a HiTrap Q HP column (GE, USA) and desalted on a HiTrap Desalting column (GE Healthcare) with desalting buffer (20 mM Tris-HCl and 150 mM NaCl, pH 8.0). Purified protein was visualized on Coomassie-stained sodium dodecyl sulfate-polyacrylamide gel electrophoresis (SDS-PAGE), and the concentration was determined using a Bradford Protein Assay Kit (Tiangen).

### Electrophoretic mobility shift assays

Fragments containing putative promotors were amplified from the cosmid 14A6 using primers listed in Table S3, and then the generated double-stranded DNA products modified by 6-carboxyfluorescein (FAM) at 5′ ends were purified with a nucleic acid purification kit (Omega, USA). The 44 bp probe (OBS_WT_) and its variants including, OBS_M1_, OBS_M2_, OBS_M3_, OBS_M4_, OBS_M5_, OBS_M6_, and OBS_M7_ (Table S3) used for electrophoretic mobility shift assay (EMSA) were generated by annealing 5′-FAM-labeled bottom strand with its respective complementary strand (OBS, MilO binding site). The labeled DNA probes were incubated with proteins in EMSA buffer (20 mM Tris-HCl, pH 8.0, 100 mM KCl, 2.5 mM MgCl_2_, 0.2 mM DTT, and 10% glycerol) in a total volume of 10 µL at 30°C for 10 min. Salmon sperm DNA was added as a nonspecific competitor for protein binding at a concentration of 0.02 µg/mL. The unlabeled probes were prepared as described above and then introduced to the system to verify the specific binding. The samples were then loaded onto 6% native polyacrylamide gels (with the ratio of acrylamide: methylenebisacrylamide being 29:1, wt/wt) and electrophoresed in 0.5  ×  TBE buffer at 15 mA for 60 min (200 bp probes, Fig. 5) or 40 min (44 bp probes, Fig. 6) in an ice bath. Gels were imaged using a Gel-Doc XR+ Imager (Bio-Rad) with an FAM fluorescence detection channel.

### DNase I footprinting assay

DNase I footprinting assay was performed as described by Wang et al. (24). To determine the precise binding site of MilO in the promoter region of *milA*, the labeled DNA probes were prepared as before. 250 ng labeled DNA probes were incubated with a range of concentrations of the recombinant protein MilO (0–10 µg) in a total volume of 40 µL at 25°C for 30 min, followed by the addition of 0.015 U DNase I and 100 nmol freshly prepared CaCl_2_ with further incubation at 37°C for 1 min. Then the reaction was terminated by adding DNase I stop solution (200 mM sodium acetate, 30 mM EDTA, and 0.15% SDS). The mixture was extracted with phenol chloroform and precipitated with ethanol. The pellets were dissolved in 30 µL of MilliQ water and analyzed with a 3730XL DNA analyzer (Applied Biosystems, USA).

### Fluorescence polarization assay

The 44 bp probe (OBS_WT_) and its variants (OBS_M1-M7_, Table S3) were used for fluorescence polarization assay. Twofold serially diluted protein solutions (5 µM starting concentration, 12 dilutions) were mixed with a final concentration of DNA probe of 5 nM in a Corning 3575 plate, using reaction buffer composed of 20 mM Tris (pH 8.0), 50 mM KCl, 2.5 mM MgCl_2_, 0.2 mM DTT, 0.02 µg/µL salmon sperm DNA, and 5% glycerol. The fluorescence polarization was detected by BioTek Synergy2 with an excitation wavelength of 485 nm and an emission wavelength of 528 nm. The K_D_ value was calculated by the binding equation Y = B_max_*X/(K_D_ +X) using GraphPad Prism 8.0.2, where Y is the ΔmP measured at a given protein concentration (X) and B_max_ is the maximum ΔmP of completely bound DNA.

### Purification and characterization of compound 1 and compound 2

Strains Δ*milN::milO* fermentations were scaled up for separation and purification of compound **1** and compound **2** with the same operations as described previously. The difference is that in the purification process, about 5 L of the fermentation broth supernatant was concentrated by rotary evaporation, then loaded onto the 500 mL macroporous adsorption resin HP-20 column (400 mm × 60 mm, m-chemical, China), washed with five column volumes of ddH_2_O and finally eluted with a gradient of 5%, 10%, and 20% methanol aqueous solution. The eluates were analyzed by HPLC using a TC-C18 column (5 µm, 250 mm × 4.6 mm; Agilent) on an Agilent 1260 Infinity series system at a flow rate of 0.8 mL min^−1^ with the following parameters: column at room temperature; solvent A, 10 mM trichloroacetic acid; solvent B, MeOH; gradient, 10% B for 30 min; detection by UV absorbance at 279 nm, the eluting fractions with the target compounds were collected, concentrated by spin-evaporation, and further purified in HPLC using a semi-preparative SB-C18 column (5 µm, 250 mm × 9.4 mm; Agilent) at a flow rate of 2 mL min^−1^ with the following parameters: column at room temperature; solvent A, 10 mM trichloroacetic acid; solvent B, MeOH; gradient, 10% B for 25 min; detection by UV absorbance at 279 nm. The collected compounds **1** and **2** were vacuum-dried to give about 4 mg and 2 mg of white powder, respectively. The 1D and 2D NMR spectra were recorded on a Bruker Avance III 700 MHz spectrometer (Billerica, MA, USA) with a BBO cryoprobe. The NMR data were acquired with Bruker spectrometers using CD_3_OD as solvent. The LC-Q-TOF-MS/MS was performed with the same parameters as that for the LC-Q-TOF-MS analysis. Target MS/MS mode was adopted for data collection. The collision energy was set to 10 eV.

### Bioinformatic analysis

The referenced protein sequences used in this study are listed in Table S4. Protein sequences were aligned by MEGA-X and visualized in ESPrit3.0 (25). Phylogenetic trees were constructed by MEGA-X. Protein structure was forecasted by alphafold2 prediction (26). Conserved motifs in the six promoter regions were identified through MEME (27).

## RESULTS

### MilO is a LuxR-family transcription regulator and is indispensable to mildiomycin biosynthesis

**Fig 2:**
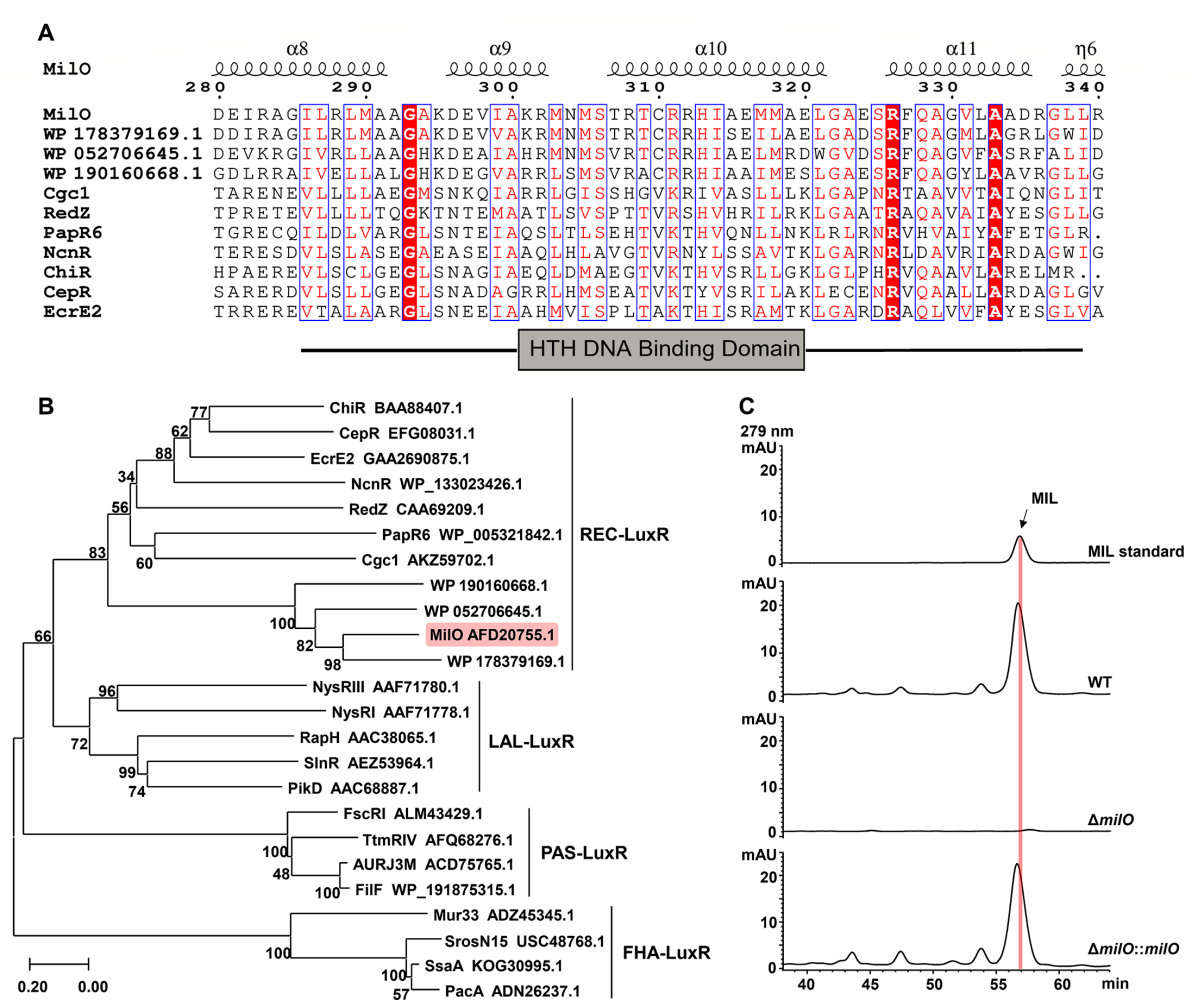
MilO is a LuxR family transcription regulator and is indispensable to mildiomycin biosynthesis. (**A**) Sequence alignment of MilO with other homologous proteins, the HTH domain was marked with the horizontal line. (**B**) Phylogenetic analysis of MilO and other LuxR-type regulators and the accession number of all proteins are listed in Table S4. REC, receiver domain. LAL, larger ATP-binding regulators of the LuxR. PAS, Per (period circadian protein), Arnt (Ah receptor nuclear translocator protein), Sim (single-minded protein). FHA, fork head-associated domain. (**C**) HPLC analysis of mildiomycin in WT (*S. avermitilis*::14A6), *milO* deletion mutant (Δ*milO*), and complementary strain (Δ*milO::milO*). The complementary *milO* is expressed under the promoter of *kasO*p*.

Previously, we discovered the BGC of mildiomycin from *Streptoverticillium rimofaciens* (on cosmid 14A6) and accomplished heterologous production in *S. lividans* with a relatively low yield (6). The bioinformatic study suggested that *milO* is the only regulatory gene in the mildiomycin BGC of *S. rimofaciens*. It has an open reading frame of 1071 bp and encodes a putative 356 amino-acid protein. MilO was predicted to be a LuxR family regulator, containing a typical LuxR-like helix-turn-helix (HTH) DNA binding domain at the C-terminus (amino acids 286–333) and an unannotated domain at the N-terminus (Fig. 2A). Among the LuxR family regulators that have been classified and characterized, MilO shows the most but moderate sequence similarity with Cgc1. It is a response regulator containing the N-terminal receiver (REC) domain and LuxR-like DNA binding domain, which regulates congocidine production in *Streptomyces ambofaciens* as an atypical orphan response factor (28). The phylogenetic analysis also demonstrated that MilO and its uncharacterized homologs clustered into the same clade with other REC-LuxR family regulators (Fig. 2B). In bacteria, REC-LuxR family regulators could form two-component regulator systems with sensor histidine kinase (SHK) to respond to intracellular or extracellular stimuli. The typical REC domain contains five conserved residues (DD, D, T, and K) that are vital for phosphorylation (29) (Fig. S1). However, some REC-LuxR family regulatory proteins function independently as atypical orphan receptors with no SHK nearby and lack two or more of the five important conserved residues, such as Cgc1, RedZ, and PapR6 (Fig. S1) (28, 30, 31). Sequence alignment analysis showed that MilO lacked the corresponding conserved amino acid residues (Fig. S1). In addition, no cognate HK genes were found within the *mil* BGC, suggesting MilO is a putative atypical orphan response regulator.

Previous gene replacement of *milO* by *aadA* totally disrupted the production of mildiomycin (6). This result led us to propose that MilO might play a positive role in mildiomycin biosynthesis. However, no genetic complementation experiment had been done to exclude the polar effect. To further investigate the role of *milO*, it was in-frame deleted using the PCR-targeting method (Fig. S2). The cosmid 14A6 (containing the entire MIL gene cluster) and 14A6Δ*milO* were then separately introduced to *Streptomyces avermitilis* by conjugation, yielding two strains *S. avermitilis* 14A6 (named as WT strain later) and *S. avermitilis* 14A6Δ*milO* (named as Δ*milO* strain later). The reason that *S. avermitilis* was selected as the heterologous host for mildiomycin production instead of *Streptomyces lividans*1326 used in our previous study is its better performance in expressing mildiomycin BGC. There was no perceptible difference in growth rate or sporulation of the WT and Δ*milO* strains on agar plates. The mildiomycin yield was then determined from shake flask fermentation cultures of both strains. HPLC analysis demonstrated that the biosynthesis of mildiomycin was abolished in the Δ*milO* strain (Fig. 2C). To exclude the polar effect as a result of the Δ*milO* mutant upon subsequent heterologous biosynthesis of mildiomycin, we performed the genetic complementation experiment. We constructed an integrative plasmid carrying *milO* under the control of the widely used *Streptomyces* strong promoter *kasO*p* on the pPM927 vector (pPM927-*kasO*p*-*milO*) and introduced it to the Δ*milO* strain. Mildiomycin production of this new strain was successfully restored and further confirmed by LC-Q-TOF-MS analyses (Fig. 2C; Fig. S3). These results clearly demonstrated that *milO* is indispensable for mildiomycin biosynthesis in heterologous hosts *S. avermitilis*, and deletion of it does not disrupt any other genes required for biosynthesis. Taken together, MilO was proposed as a positive regulator in the mildiomycin biosynthesis.

### Overexpression of *milO* enhances mildiomycin production

**Fig 3:**
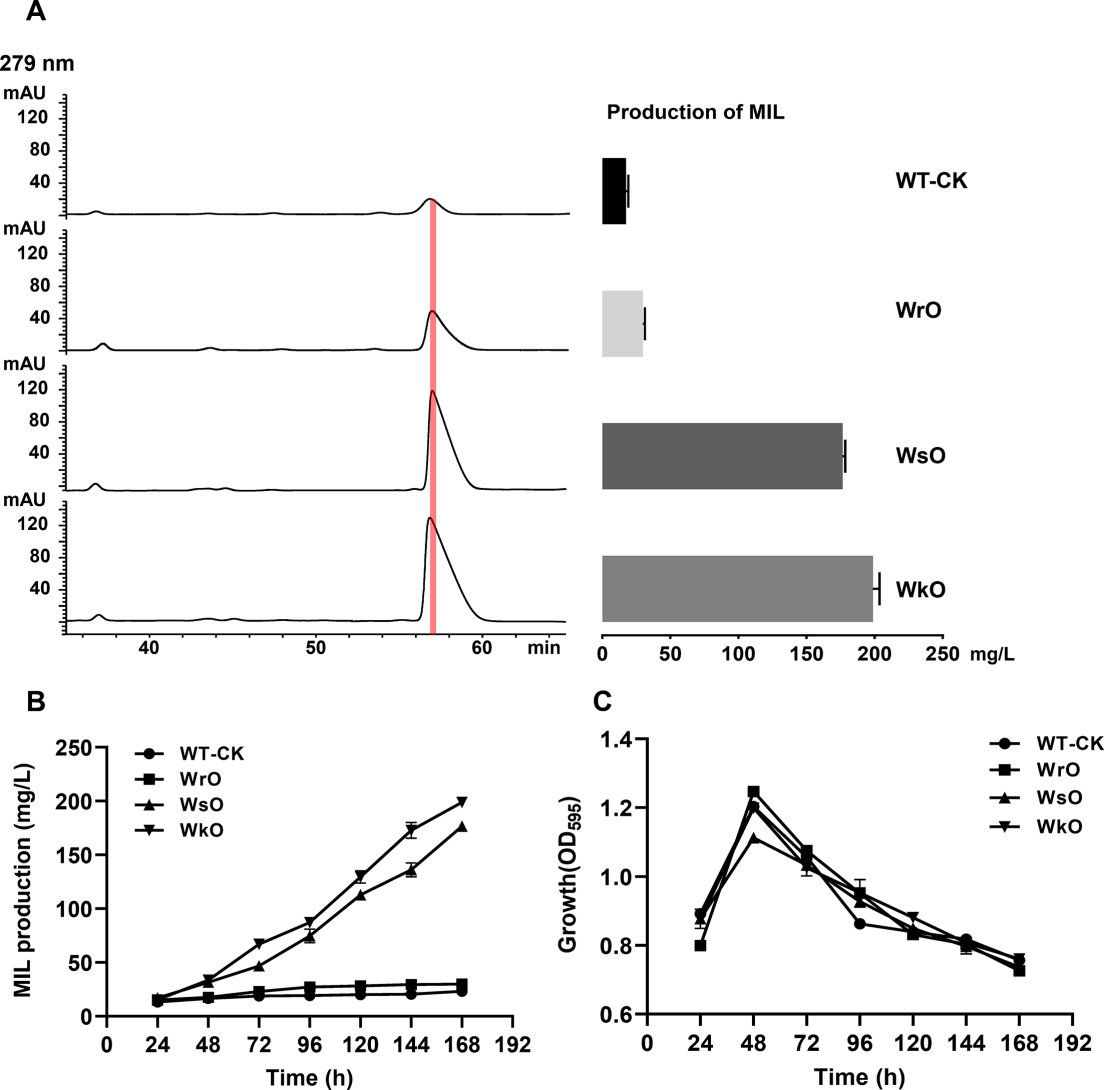
Exploring the effect of *milO* overexpression on mildiomycin titer. (**A**) HPLC analysis of mildiomycin production in WT-CK (WT::pPM927), WrO (WT::pPM927-*rpsJ*p-*milO*), WsO (WT::pPM927-*SP44-milO*), and WkO (WT::pPM927-*kasO*p*-*milO*) (left); titers of mildiomycin from three replicative fermentations for each of strains were quantified (right). All strains were cultured in a fermentation medium for 7 days. (**B**) Time-course production of mildiomycin for three producer strains. (**C**) Time-course biomass of three mildiomycin producers. The OD_595_ values determined by the diphenylamine colorimetric method were proportional to the biomass.

To further confirm the role of MilO as a positive regulator in mildiomycin biosynthesis and increase the production of mildiomycin, we overexpressed MilO in the WT strain. Besides the aforementioned complementary plasmid pPM927-*kasO*p*-*milO*, the other two *milO* overexpression integrative plasmids under the control of the host native promotor *rpsJ*p and engineered promotor *SP44* were constructed (pPM927-*rpsJ*p-*milO* and pPM927-*SP44-milO*). Then the three plasmids containing the *milO* gene were transferred to the WT strain using conjugation to give rise to three engineered strains, named WrO (*milO* under the control of *rpsJ*p), WsO (*milO* under the control of *SP44*), and WkO (*milO* under the control of *kasO*p*) strains. PCR assays confirmed that the recombinant plasmids were successfully integrated into the chromosome of the WT strain (Fig. S4). As a control, the wild-type strain was conjugated with the empty pPM927 (Fig. S4). The yield of the WrO strain using the host promoter increased continuously from 24 h to 96 h and then tended to be constant, while the yield of WT only increased significantly from 24 h to 48 h (Fig. 3B). At the end of the fermentation, the WrO strain produced 50% more mildiomycin than the latter (Fig. 3A). Interestingly, the WsO and WkO strains showed a continuous increase in mildiomycin production over 7 days (Fig. 3B). More importantly, the mildiomycin production of the WsO and WkO strains were 7.5 and 10.2 times to that of the control strain, and the WkO strain reached a maximal final titer of 200 mg/L (Fig. 3A). To explore the relationship between the increased mildiomycin production and cell growth, the growth curves of the four strains were measured (Fig. 3C). Overexpression of MilO and integration of empty vector pPM927 into the *S. avermitilis* chromosome had little effect on the cell growth indicating the increased mildiomycin production in the recombinant strains was not due to the change of cell growth (Fig. 3C). The results indicated that the yield of mildiomycin could be greatly increased by overexpression of *milO*.

**Fig 4:**
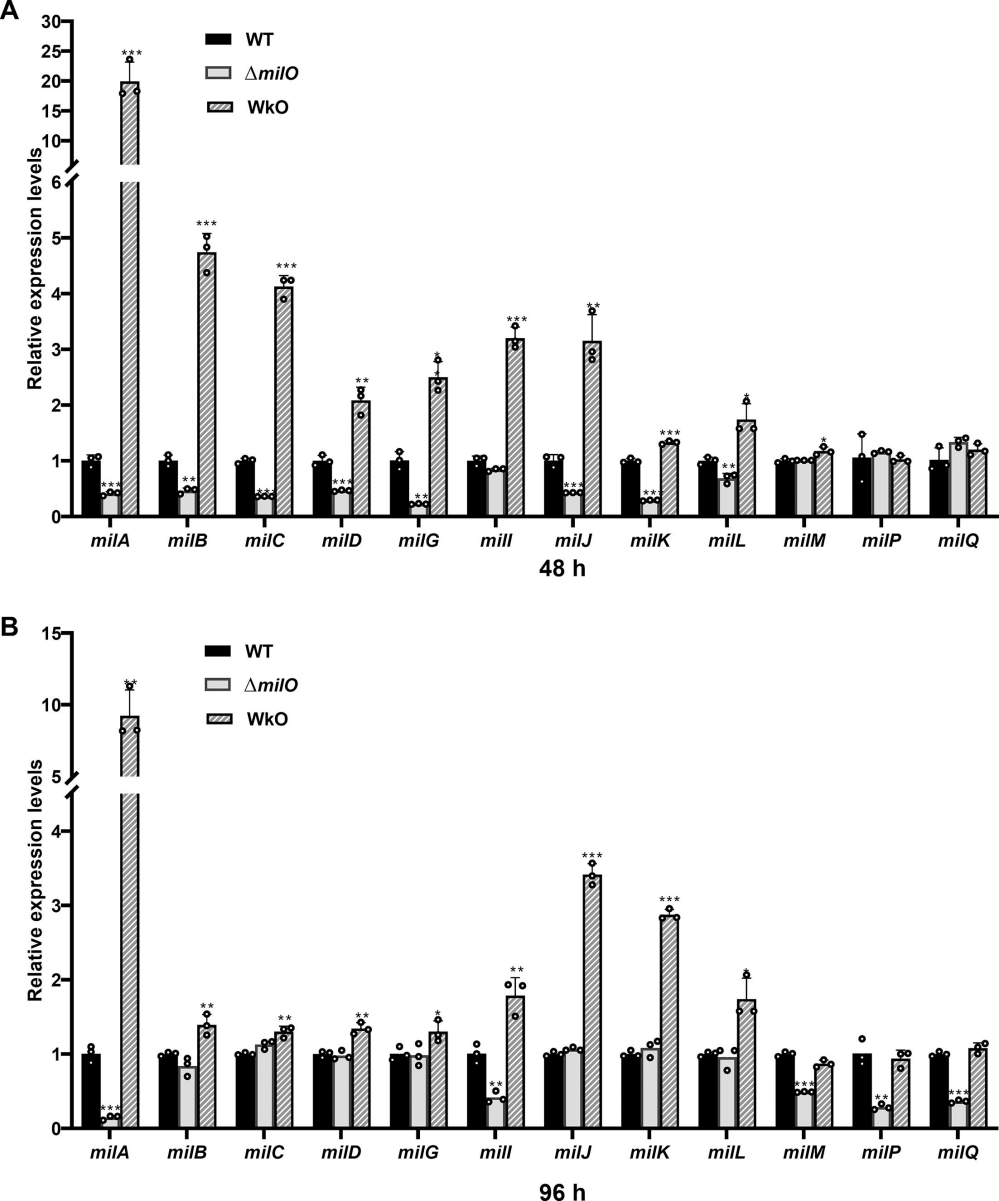
qPCR-based comparison of expression of *mil* genes in strains with varied expression of MilO. The cells were harvested from the fermentation broth after 48 h (**A**) and 96 h (**B**). Error bars were calculated by measuring the standard deviations of the data from three replicates of each sample. *, **, and *** indicate statistically significant results (*P*-value < 0.05, *P*-value < 0.01 and *P*-value < 0.001, respectively).

We speculated that MilO might affect the production of mildiomycin by positively regulating the transcription of the structural genes in the *mil* cluster. To confirm this hypothesis, the influence of *milO* on the transcription of representative *mil* genes was analyzed in three strains: WT, Δ*milO,* and WkO. The strains were grown in a fermentation medium, and the total RNAs used as the template for cDNA synthesis were extracted from mycelium harvested after 48 h and 96 h. Quantitative real-time PCR (qPCR) analysis was used initially to assess the *mil* gene transcription levels in all three strains, and *hrdB* was employed as an internal control for each cDNA template. As shown in Fig. 4, the deletion of *milO* drastically reduced the transcription of the genes from *milA* to *milQ* in the BGC of MIL to a trace level in comparison with the transcription levels of the *mil* genes in the WT strain. On the other hand, the transcription level of *milA* dramatically increased (20 times at 48 h and 9.2 times at 96 h in comparison with the WT strain) and the other representative *mil* genes increased to varying degrees in the WkO strain. Thus, the qPCR results suggest that MilO is a pathway-specific positive regulator of mildiomycin biosynthesis.

### MilO protein binds specifically to the promoter regions of *milA*

**Fig 5:**
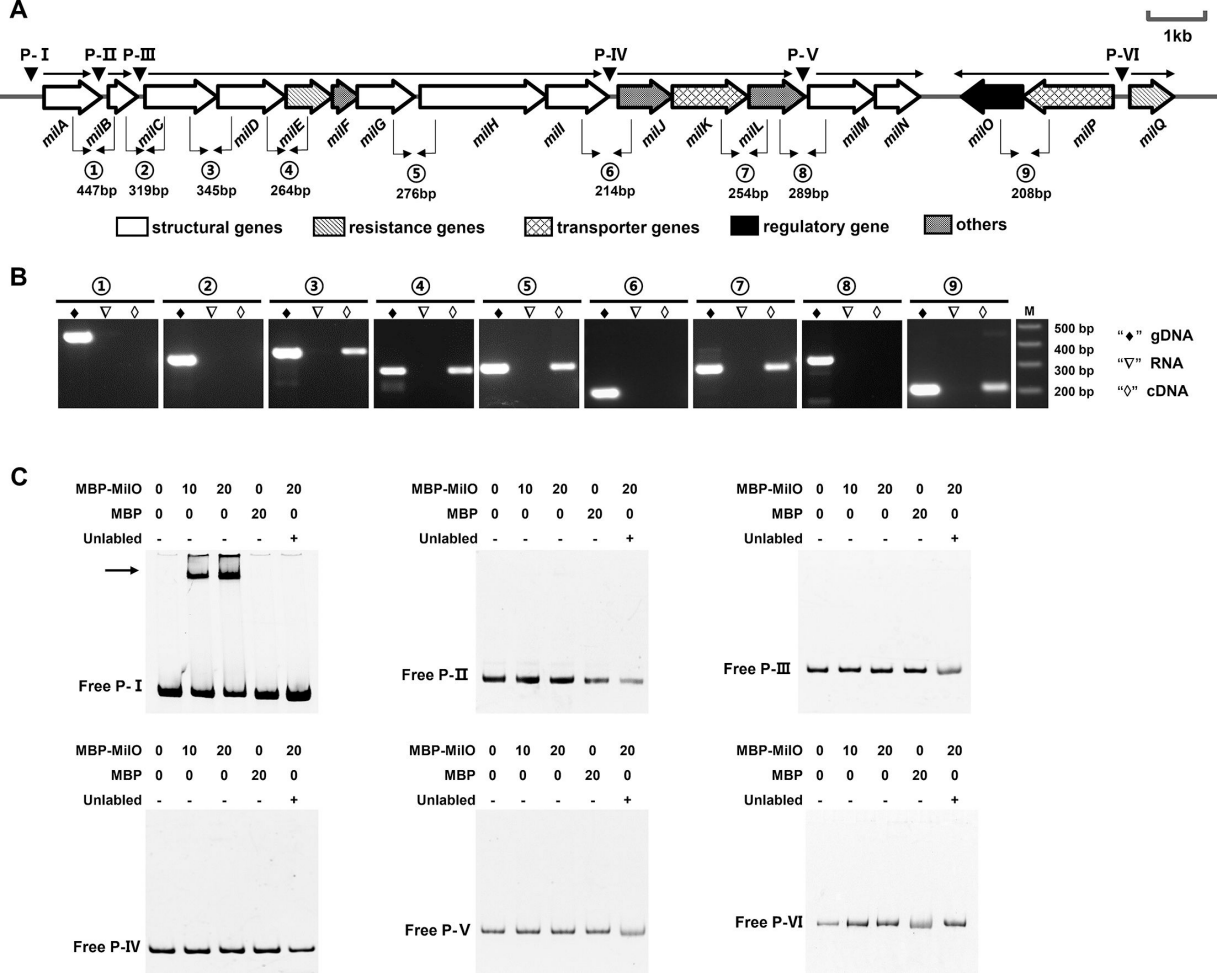
EMSAs to examine the binding of MilO to six promotors of transcriptional units. (**A**) *mil* gene organization and seven putative transcriptional units that are determined by RT-PCR analysis in panel B. The arrows show their orientation; the promoters for each of the transcriptional unit were indicated with P-I, P-II, P-III, P-IV, P-V, and P-VI (black triangles); the circled numbers denote the PCR fragment across two neighboring *orfs*. They are the same as those in panel **B**, which shows the RT-PCR analysis of the transcriptional units. The cDNA was generated by reverse transcription of total RNA samples, genomic DNA, and total RNA of WT were used as control. (**C**) 3 pmol FAM-labeled DNA probes for each promoter was incubated with 10 pmol or 20 pmol recombinant MilO at room temperature for 10 min. The unlabeled probe of 60 pmol (the last lane for each gel) was used as the control to demonstrate specific and competitive binding with MilO. 0.2 µg salmon sperm DNA was included in each reaction. The arrow indicates the shifted bands of the protein-DNA complexes. MBP, MBP-His_8_; MBP-MilO, MBP-His_8_-MilO.

To determine the regulation mode of MilO and investigate whether MilO could directly regulate the expression of these *mil* genes, we first characterized the transcription units of the *mil* gene cluster. Using RNA extracted from the mycelium cultured for 24 h as a template, we analyzed the co-transcriptional profiles of 17 genes by RT-PCR using primers listed in Table S3. Each pair of adjacent genes with gaps was examined, and genes in the same direction with overlapping regions were assumed to be one co-transcribed unit by default (Fig. 5A). As shown in Fig. 5B, we determined that the *mil* gene cluster consists of seven transcription units, including three monocistronic transcription unit (*milA, milB,* and *milQ*) and four multicistronic (*milC-D-E-F-G-H-I, milJ-K-L, milM-N,* and *milO-P*). Moreover, our co-transcript results corresponded with the qPCR, that is, the expression levels of genes on the same cistron remained consistent.

As detected by qPCR, we observed a significant increase in the expression levels of some *mil* genes, especially *milA* and *milB*, in strain WkO. To determine whether MilO directly binds to the promoter regions and regulates the expression of these *mil* genes, EMSAs were performed. To this end, we attempted to purify the recombinant MilO. Unfortunately, despite considerable efforts, we could not obtain soluble recombinant MilO with N-terminal His_6_-tag in *E. coli* BL21 (DE3). Thus, MilO was fused with the maltose binding protein (MBP) into a chimeric MBP-His_8_-MilO with a predicted molecular weight and purified using nickel affinity chromatography. Purified MBP-His_8_-MilO was then used to carry out EMSA experiments and MBP-His_8_ was used as a control (Fig. S5). Based on the results of co-transcripts, six 6-carboxyfluorescein (FAM)-labeled probes corresponding to six promoters for *mil* genes, in which probe VI is shared by two divergent promoters of MilP and MilQ (Fig. 5A). EMSAs demonstrated that MBP-His_8_-MilO could retard the probe for the promoter region of *milA* while MBP-His_8_ could not, demonstrating the interaction between MilO and the promoter region of *milA* (Fig. 5C). When excessive unlabeled *milA* probe was added to the binding reactions, the retarded band for *milA* probe disappeared (decreased), strongly suggesting that MilO binds specifically to the promoter region of *milA* (Fig. 5C). For the rest probes, no evident retarded bands were obtained, indicating MilO could not bind to these promoters directly (Fig. 5C).

In other words, only *milA* among the *mil* gene cluster was directly controlled by MilO, the other six operons/genes were positively and indirectly controlled by MilO. MilA was demonstrated as a cytidine 5′-monophosphate (CMP) hydroxymethylase to provide a precursor to mildiomycin in our previous study and initiate mildiomycin synthesis (7). Both qPCR and EMSA assays suggested that MilO increased the production of mildiomycin mainly by increasing the efficiency of the initial step of mildiomycin biosynthesis through upregulating *milA* expression, which was also a rate-limiting process in the product synthesis.

### MilO binding site contains three imperfect direct repeats in the *milA* promoter region

**Fig 6:**
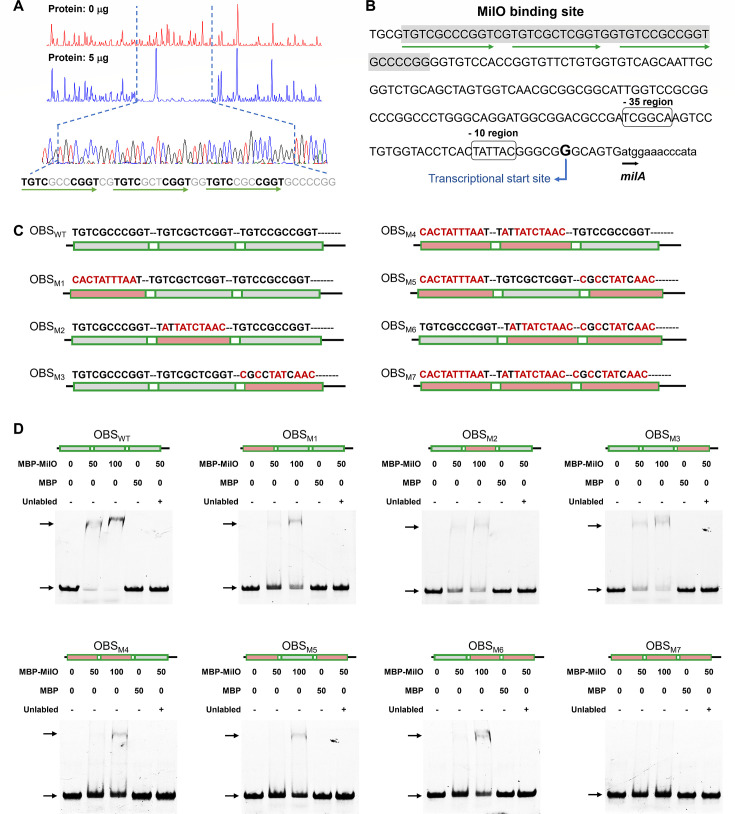
DNA-binding properties of MilO targeting the MilA promoter region. (**A**) DNase I footprinting analysis of MilO in the promotor of MilA (probe P-I). The sequence of the protected region is indicated below the electropherograms, and the three 11 bp direct repeats sequence was shown in green. (**B**) Nucleotide sequence of the MilA promoter region. The predicted transcription start sites (TSS) are bolded and enlarged, the putative −10 and −35 regions are boxed out. (**C**) Schematic diagram to show the sequence of eight probes with various mutations at three direct repeats. The mutated nucleotide sequences are shown in red. The three direct repeat sequences are shown in gray and the spacers in white. The variants are shown in pink. Sizes are not proportional. (**D**) EMSAs of MilO binding to OBS_WT_ and its variants (OBS, MilO binding site). The 44 bp FAM-labeled DNA oligos (0.5 pmol) were incubated with increasing concentrations of recombinant MilO (lane 2, 25 pmol; lane 3, 50 pmol). Lane 1, negative control without MilO; lane 4, MBP alone with 0.5 pmol labeled probe; lane 5, 50 pmol unlabeled probe was added to the reaction system for lane 2. 0.2 µg Salmon sperm DNA was included in each reaction mixture. MBP MBP-His_8_; MBP-MilO, MBP-His_8_-MilO.

Next, we turned our attention to determine the precise binding site of MilO in the promoter region of *milA*. DNase I footprinting experiments were performed using a 6-carboxyfluorescein (FAM)-labeled probe. It was revealed that there is a MilO binding site (OBS), a continuous 44 bp DNA sequence (5′-TGTCGCCCGGTCGTGTCGCTCGGTGGTGTCCGCCGGTGCCCCGG-3′, OBS_WT_) that includes three imperfect 11 bp direct repeats (DRs; underlined), which are located 142 bp upstream of the transcription start site of *milA* (Fig. 6A and B). To examine the role of each DR in the binding with MilO, seven probes derived from OBS_WT_ were designed for the DNA-affinity assays. These probes were identical to OBS_WT_ except that the sequence of DRs was replaced with unrelated bases to various degrees (Fig. 6C). The EMSAs using these probes by MilO showed that the intensity of the shifted bands for OBS_M1_, OBS_M2_, and OBS_M3_, on each of which there is one DR was mutated, decreased as compared to that for the OBS_WT_ (Fig. 6D). The shifted probes for OBS_M4_, OBS_M5_, and OBS_M6_, on each of which two DRs are mutated, further decreased. Notably, probe OBS_M7_, which lost three DR elements, exhibited a complete disappearance of the shifted band (Fig. 6D). In accordance with the results of EMSAs, the binding affinity of MilO to these probes was further quantified by the fluorescence polarization assays (Fig. S6A). Compared to the OBS_WT_, mutation of one DR resulted in a ~ 3-fold decrease in binding affinity, and mutation of two DRs resulted in a ~ 14- to 19-fold decrease while mutation of three DRs led to no detection of the affinity by FP assays (Fig. S6B). These data suggest that each DR plays a role in the specific binding with MilO. Then, we used the binding sequence of 44 bp as a query to search for potential binding sites of MilO in the genome of the heterologous host *S. avermitilis*. No similar sequence was found, indicating that MilO functions as a pathway-specific regulator in the *mil* BGC and is unlikely involved in cell growth and other metabolic activities when *mil* BGC was heterologously expressed in *S. avermitilis*. Taken together, our results demonstrated that MilO can activate the transcription of *milA* through direct interaction with the three imperfect direct repeats in the *milA* promoter region.

### Overexpression of *milO* in *milN* mutant results in accumulation of MIL intermediates

**Fig 7:**
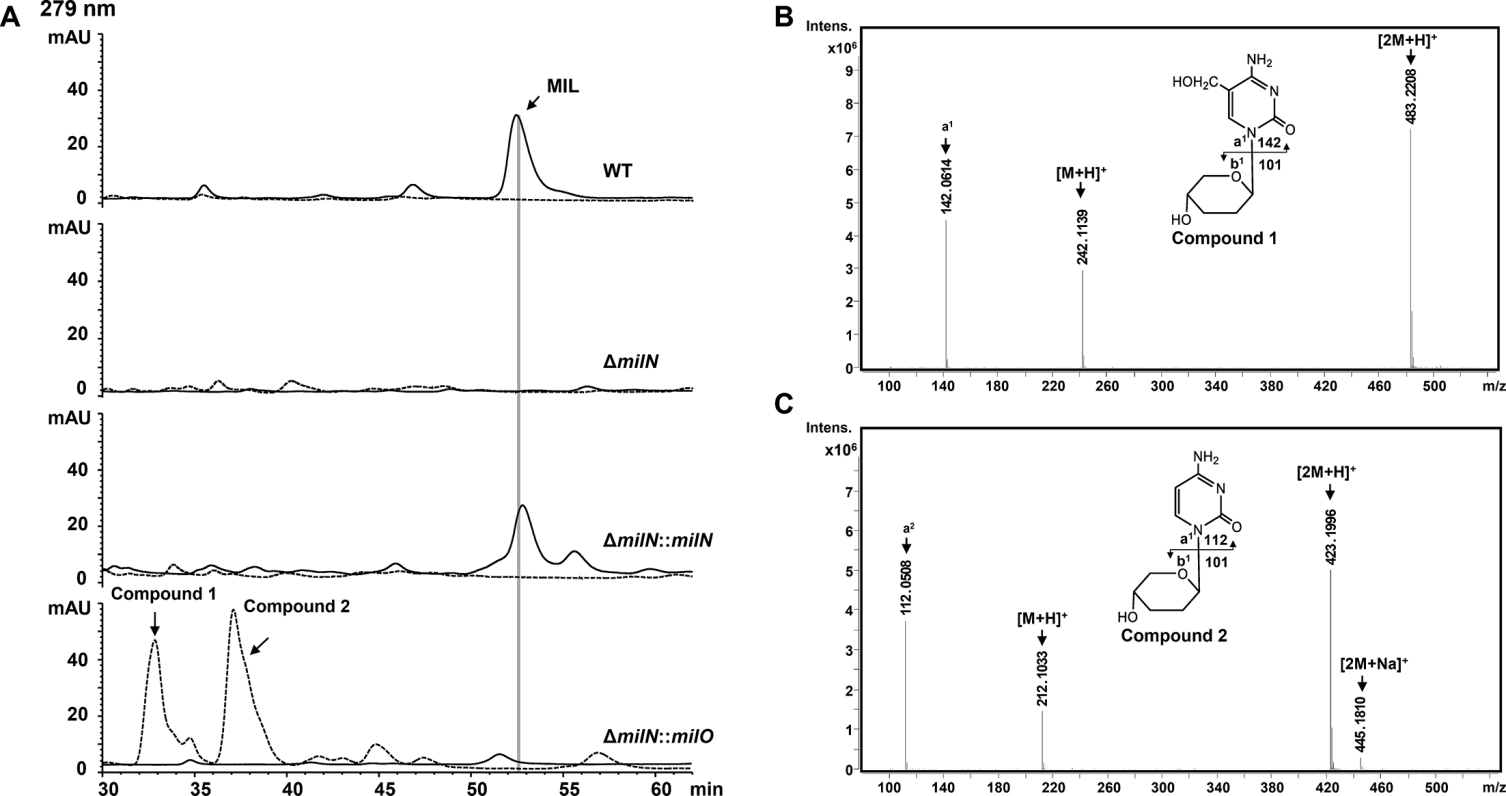
Overexpression of MilO in *milN* mutant produces two cryptic shunt products. (**A**) HPLC analysis of the products of *Streptomyces avermitilis*-derived mutants. WT is *Streptomyces avermitilis* expressing the complete mildiomycin biosynthetic gene cluster on integrative cosmid 14A6. The sample after resin purification was shown in a solid line, and the flowthrough during purification was indicated by a dashed line. (**B, C**) The LC-Q-TOF-MS analysis (positive model) of compound **1** and compound **2**. Both compounds were collected from the extract of Δ*milN::milO*. Compound **1**: [M + H]^+^ Cal. 242.1135, Obs. 242.1139, 1.65 ppm Error; Compound **2**: [M + H]^+^ Cal. 212.1030, Obs. 212.1033, 1.41 ppm Error. The inferred structural formulas are shown above.

Despite the commercial application of mildiomycin, its biosynthetic pathway has not been fully elucidated. The failure to get related metabolites of MIL biosynthesis in different mutant strains is a major obstacle. With the significant increase of MIL production in the *milO* overexpression strain, we anticipate a similar effect when MilO is overexpressed in the *mil* structural gene-deficient strain. *milN* is predicted to encode a dihydrodipicolinate synthase family protein catalyzing the uploading of the modified arginine side chain (Fig. 1) (6). However, its function and the timing of the modified arginine side chain have not been determined experimentally. To this end, an in-frame Δ*milN* deletion mutant was obtained. The resultant mutant strain lost the ability to produce MIL (Fig. 7A). The potential related products accumulated in the Δ*milN* mutant may lack the positively charged side chain and cannot bind to the cation resin. Thus, both the fraction recovered from cation resin and the flowthrough of the fermentation broth of the Δ*milN* mutant were analyzed by HPLC (Fig. 7A). No obvious accumulations of related compounds could be detected in the fermentation broth of the Δ*milN* mutant strains (Fig. 7A). Complementation experiments confirmed that the loss of MIL in Δ*milN* strain was caused by *milN* deletion (Fig. 7A).

Then, *milO* under the control of *kasO*p* was introduced into the Δ*milN* strain. Interestingly, the resultant strain accumulated two products (compounds **1** and **2**) with similar UV spectra (Fig. 7A). Through Q-TOF-MS analysis, the molecular formula for compounds **1** and **2** was predicted to be C_10_H_15_N_3_O_4_ ([M + H]^+^ m/z: Obs. 242.1139, Cal. 242.1135, 1.65 ppm Error) and C_9_H_13_N_3_O_3_ ([M + H]^+^ m/z: Obs. 212.1033, Cal. 212.1030, 1.41 ppm Error), respectively (Fig. 7B and C). MS/MS fragmentation analysis showed that compounds **1** and **2** have an identical fragment (m/z: 101. 0598). Besides, compound **1** has a daughter ion at m/z 142, corresponding to HMC moiety, and compound **2** has a daughter ion at m/z 112, corresponding to cytosine (Fig. S7) (7).

To further characterize their structures, compounds **1** and **2** were purified from large-scale fermentation and the structures were revealed by NMR spectra (Tables S5 and S6; Fig. S8 through S21). For compound **1**, the ^1^H NMR spectrum showed resonances for 11 protons, and the ^13^C NMR and HSQC data assigned 10 carbons (Table S5; Fig. S8, S9, and S12). In the HMBC spectrum, the aromatic hydrocarbon proton (H-6) correlated to the C-1′, suggesting the connection of the pyrimidine moiety to the pyranose moiety (Fig. S13). The continuous COSY correlations of H-1′/H-2′/H-3′/H-4′ and the HMBC cross-peaks of H-1′ with C-3′, C-5′, and H-5′ with C-3′, C-4′ confirmed the 2, 3-dideoxy-arabinopyrano structure (Fig. S12 and S13). The presence of NOESY cross-peaks between H-1′ and H_b_-5’, H_a_-5’ and H-4′, and the absence of peak between H-1′ and H-4′ suggested the D-configuration of **1** (Fig. S14). The NMR analysis combined with Q-TOF-MS/MS data demonstrate that the structure of compound **1** is 1-α-(2, 3-dideoxy-D-arabinopyranosyl)−5-hydroxymethyl-cytosine (Fig. 7B; Table S5; Fig. S8 through S14). A comparison of the ^13^C NMR spectrum showed that compound **2** had one less methylene at δ_C_ 57.9 than **1** (Fig. S9 and S16). NMR data analysis confirmed the structure of compound **2** to be a known compound, named pentopyranine B (PPNB) (Table S6; Fig. S14 through S20) (32, 33). Accordingly, compound **1** was named 5-hydroxymethyl-pentopyranines B (HM-PPNB). As PPNB was a shunt product of blasticidin S biosynthesis previously isolated from the fermentation broth of *Streptomyces grisechromogenes,* HM-PPNB was therefore proposed as a shunt product in MIL biosynthesis (Fig. 1; Fig. S22) (32). The proposed function of MilN is related to the uploading of the modified arginine in MIL biosynthesis (6). Accumulation of **1** and **2** lacking any side chain of MIL in *milN* mutant stains indicates that the modified arginine might be loaded onto the MIL skeleton earlier than serine (Fig. 1). This result demonstrated that the overexpression of *milO* not only enhances the production of mildiomycin in the WT strain but also increases the accumulation of related metabolites in the mutant strain.

## DISCUSSION

The nucleoside antibiotics show potent antifungal activity and can be used to treat powdery mildew. Although it was isolated from *Streptoverticillium rimofaciens* in 1978, and manufactured by Takeda Chemical Industries Ltd in 1994, its biosynthetic pathway and regulation mechanisms have yet to be fully characterized (3). The production of mildiomycin in its original strain is low and unstable. Our previous studies revealed that structural genes *milA*, *milB*, and *milC* are involved in the synthesis of C2′, C3′-didehydrated pyranose ring with hydroxymethyl cytosine (6–9). In comparison, little is known about the mechanisms of the following uploading of the two side chains. Cosmid 14A6 containing *mil* gene cluster was successfully cloned and expressed in heterologous hosts *Streptomyces lividans* and *Streptomyces avermitilis*. However, the production of MIL was very limited, and further development is needed to improve the production. Therefore, this study aims to improve MIL production and help us to further understand the biosynthetic pathways of MIL by exploring the regulatory mechanisms of MIL. By combining the use of genetic and EMSA characterization, a LuxR protein, MilO, has been identified as a pathway-specific positive regulator in mildiomycin biosynthesis.

*Streptomyces*, the largest genus of Actinobacteria, are rich resources for producing a large number of antibiotics, as well as many other natural products (34). In the past decade, with the help of fast and inexpensive genome sequencing techniques increasing *Streptomyces* genomic sequencing data have become available (34). Considering that a single *Streptomyces* genome possesses 20–30 different BGCs with known or unknown products, the tremendous amount of genomic information indicates that the *Streptomyces* has much more potential to produce natural products for greater diversities than originally expected (35). However, the production of many natural products is low and many BGCs are even silent under normal cultural conditions (35).

Generally, the onset and the level of the production of natural products are stringently regulated by complex transcriptional regulatory cascades. To date, more than 50 families of regulators (e.g., the TetR family, GntR family, LysR family, and LuxR family) have been reported (36). They can be grouped into global regulators and pathway-specific regulators according to their biological function (37). The global regulators may not be linked to specific biosynthetic gene clusters and usually control several metabolic pathways (37). Pathway-specific regulators are usually encoded by the genes located inside the BGCs of natural products and activate or repress the transcription of the neighboring biosynthetic genes (37). Transcriptional engineering represents a practical and effective approach to enhance the production of target natural products. At present, many of these complex regulatory networks have not been fully illustrated and, in turn, impede the discovery and commercialized development of natural products in *Streptomyces*.

LuxR proteins are a large family of transcriptional regulators widely distributed in microorganisms (38). It was first reported and well known as a transcriptional regulator in quorum-sensing circuit of the symbiotic organism *Vibrio fischeri* (39). Since then, LuxR family proteins are involved in the regulation of many other biological processes including biosynthesis of secondary metabolites (17, 40, 41). They are usually characterized by a helix-turn-helix (HTH) domain, named LuxR, responsible for the DNA binding (38). LuxR family proteins comprise widespread and functionally diverse transcription regulators and are mainly divided into two groups according to domain architectures: single-domain and multiple-domain LuxR regulators (38). In addition to the LuxR domain, multiple-domain LuxR regulators usually include signal-binding domains at the N-terminus that are responsible for signal perception and transduction (38). Various signal-binding domains have been identified, such as the REC domain, PAS domain, AAA domain, FHA domain, and CHD domain (38). LuxR regulators are mostly pathway-specific in the biosynthetic gene clusters of natural products and function as activators by direct or indirect activation of the genes inside the BGCs (36). Many LuxR regulators have been characterized, for example, the RedZ is involved in undecylprodigiosin biosynthesis in *Streptomyces coelicolor* and Cgc1, involved in netropsin biosynthesis in *Streptomyces ambofaciens* (28, 30). Although LuxR proteins usually function as pathway-specific activators in natural product biosynthesis, a few are involved in two or more BGCs, such as TtmRIV, a cluster-situated PAS-LuxR as an activator in the tetramycin BGC and also as a repressor for nystatin A biosynthesis in *Streptomyces ahygroscopicus* (42). With the knowledge of how these regulators are involved in natural product biosynthesis, tremendous successful cases have been reported about the increase in the production by manipulation of regulators (36).

There are few studies about the transcriptional regulation of MIL biosynthesis. In this study, MilO is identified as the only regulator in *mil* BGC belonging to the LuxR family. We demonstrated the importance of MilO in mildiomycin biosynthesis by genetic deletion and complementation experiments. In the *milO* deletion mutant, the production of mildiomycin is completely abolished, and the production was restored after the *milO* complementation. The overexpression of MilO driven by three different promoters including *rpsJ*p from *S. avermitilis* as well as two commonly used highly active engineered promoters all enhance the production of mildiomycin significantly. The qPCR experiments demonstrated that the transcriptional levels of all the *mil* structural genes were increased to varying degrees in the engineered strains overexpressing MilO. Interestingly, a conserved sequence 5′-GGACGDSKCG-3′ (D: A, T, or G; S: A, C, or G; K: T or G) could be identified through gene scanning in the upstream regions of six operons of *mil* BGC (Fig. S23). To our surprise, EMSA experiments indicated that the MilO can only directly bind the promoter region of *milA* notably. DNase I footprinting experiments revealed a continuous 44 bp DNA sequence as the MilO binding site. Interestingly, the binding site contains three imperfect direct repeats of 5′-**TGTC**NNN**CGGT**-3′ separated by two nucleotide spacers. Each DR element contributes to MilO binding to this region and the destruction of either DR will decrease the binding of MilO. Such a DR orientation in the binding regions of regulators has been reported for other regulatory proteins (43, 44). PimR and PnR2, two SARP-LAL family regulators, are proposed to bind three heptameric direct repeats as monomers to regulate the production of the antifungal pimaricin in *Streptomyces natalensis* and phoslactomycin in *Streptomyces platensis strain* SAM-0654 (43). The importance of three DRs for MilO binding implied that MilO might function as monomers in the binding with each DR. Interestingly, MilO can also positively regulate several other genes in MIL BGC, although there are no evident interactions between MilO and other promoters in *mil* BGC. Previous studies have shown similar results that some pathway-specific regulatory proteins can regulate the transcription of genes indirectly. For instance, CepR involved in the biosynthesis of cephamycin C regulates the transcription of most cephamycin C biosynthetic genes while it only interacts with the *cefD-cmcI* bidirectional promoter (45); RimR2, a positive specific-pathway regulator of rimocidin biosynthesis in *Streptomyces rimosus* M527, regulates the rimocidin biosynthesis by influencing the transcriptional levels of all *rim* genes and only binds to the promoter regions of *rimA* and *rimC* (17). Taken together, only *milA* among *mil* biosynthetic operons could be directly regulated by MilO, which is consistent with that the increase in transcription level of *milA* is much higher than that of other *mil* structural genes, and other operons might be regulated by MilO indirectly. MilA plays an important role in the initiation of MIL biosynthesis as a CMP hydroxymethylase to pull cytidine 5′-monophosphate into MIL biosynthesis.

In our previous studies, we did not detect any evident accumulations of the intermediates or shunt products in the mutant strains with the deletion of structural genes except for the Δ*milA* and Δ*milG* mutants, hindering further biosynthetic studies of mildiomycin (6). We speculated that the amount of the accumulated related metabolites was too low to be detected. Identification of MilO as a pathway-specific positive regulator provides us with a new feasible strategy. Fortunately, when MilO was overexpressed in the *milN*-deletion strain, two related products were accumulated. In comparison, these two products could not be detected in the original *milN*-deletion mutant strain. The proposed function of MilN is the transfer of the modified arginine side chain onto the skeleton of MIL (Fig. 1). The structural determination of the two compounds accumulated in the Δ*milN::milO* strain revealed that they are shunt products lacking both side chains during MIL biosynthesis. Although they could not be directly used as the potential substrates for the following enzymatic assays for MilN, the structural characters still provide important clues for the timing of uploading of two amino acids during MIL biosynthesis for the first time. MilN catalyzes the transfer of the modified arginine side chain prior to the serine side chain uploading. In conclusion, we identified that the fermentation titer was dramatically improved by overexpression of the native cluster-situated pathway-specific regulator MilO, which plays a direct regulatory role by specifically binding to the promoter region of *milA*. This study provides a basis for further studies on the yield improvement and biosynthetic pathway of mildiomycin.

## Data Availability

The underlying data are available in the article and in its supplemental material.

## SUPPLEMENTAL MATERIAL

Supplemental material aem.01654-24-s0001.pdf Tables S1 to S6; Figures S1 to S23.

ASM does not own the copyrights to Supplemental Material that may be linked to, or accessed through, an article. The authors have granted ASM a non-exclusive, world-wide license to publish the Supplemental Material files. Please contact the corresponding author directly for reuse.
