# Supplementary material for: Positive regulation of a LuxR family protein, MilO, in mildiomycin biosynthesis
Source: Appl Environ Microbiol. 2024 Dec 23;91(1):e01654-24. doi: 10.1128/aem.01654-24 (PMC11784345; doi:10.1128/aem.01654-24)
Supplement: Supplemental material — Tables S1 to S6; Figures S1 to S23. [file aem.01654-24-s0001.pdf]

Positive regulation of a LuxR family protein, MilO, in mildiomycin biosynthesis

AUTHORS

Zhiyu Li[1], Yuli Wang[1], Chen Lin[1], Yu Wen[1], Zixin Deng[1], Ming Jiang[1]* and Xinyi He[1]*

[1] State Key Laboratory of Microbial Metabolism, Joint International Research Laboratory of Metabolic & Developmental Sciences, School of Life Sciences & Biotechnology, Shanghai Jiao Tong University, 800 Dongchuan Road, Shanghai 200240, People's Republic of China

*To whom correspondence should be addressed. Tel: (86)-2162932943; Fax: +86 21 6293 2418; E-mail: xyhe@sjtu.edu.cn or jiangming9722@sjtu.edu.cn

Table S1 Annotation of proteins referred in Figure 1 involved in mildiomycin biosynthesis

Protein	Accession	Description	Reference
MilA	ACA14348.1	CMP 5-hydroxymethylase	(1)
MilB	ACA14349.1	nucleotide hydrolase	(1)
MilC	AFD20743.1	cytosyl-glucuronic acid/hydroxymethyl cytosyl-glucuronic acid synthase	(2)
MilD*	AFD20744.1	degT/dnrJ/eryC1/strS aminotransferase	(2)
MilG*	AFD20747.1	radical SAM superfamily, cytosylglucuronate decarboxylase	(2)
MilM*	AFD20753.1	pyridoxal phosphate–dependent arginine oxidases	(3)
MilN*	AFD20754.1	dihydrodipicolinate synthetase family protein	(2)
MilI*	AFD20749.1	acyl carrier protein	(2)
MilH*	AFD20748.1	BlsK-like protein, tRNA-dependent aminoacyltransferase	(2)

“*” protein function has not been experimentally verified.

Table S2 Strains and plasmids used in this study

Strains or plasmid	Description	Reference
Strains		
Escherichia coli ET12567/pUZ8002	*rec*F, *dam*, *dcm*, *hsdS*, Cml^r, Str^r, Tet^r, Km^r	(4)
BW25113/pIJ790	RepA101(ts), *araBp-gam-be-exo*, *AraC,* RepA101(ts)*,* Cml^r	(5)
BL21 (DE3)	F^-, *ompT*, $hsdS_b$ (r_b^-, m_b^-), *gal*, *dcm*, (DE3), pLysE	Novagen
Streptomyces avermitilis NRRL 8165	Wild type	Laboratory stored
WT	*Streptomyces avermitilis* NRRL 8165 containing cosmid 14A6 (see below plasmids)	This study
Δ*milO*	WT with *milO* in-frame deletion	This study
Δ*milO*::*milO*	WTΔ*milO* carrying *kasO*p*-*milO*	This study
Δ*milN*	WT with *milN* in-frame deletion	This study
Δ*milN*::*milN*	WTΔ*milN* carrying *kasO*p*-*milN*	This study
Δ*milN*::*milO*	WTΔ*milN* carrying *kasO*p*-*milO*	This study
WT-CK	WT carrying pPM927(see below plasmids)	This study
WkO	WT carrying *kasO*p*-*milO*	This study
WsO	WT carrying *SP44-milO*	This study
WrO	WT carrying *rpsJ-milO*	This study
Plasmids		
14A6	Integrative cosmid harboring the biosynthetic gene cluster of mildiomycin, pSET152 derivative, *aac(3)IV*	(1)
pPM927	Integrative and conjugative vector used in	(6)

	actinomycetes with *aadA* and *tsr*	
pPM927-1	pPM927 with *kasO*p*-*milO*	This study
pPM927-2	pPM927 with *SP44-milO*	This study
pPM927-3	pPM927 with *rpsJ-milO*	This study
pPM927-4	pPM927 with *kasO*p*-*milN*	This study
pSJ8	pET43.1a derivative, MBP tag, *bla*	Dr. Jiahai Zhou

Table S3 Primers used in this study

Primer name	Sequence	Usage
14A6-KO-F	5'-cccgcgccgccaggcgcgggaaaccccacggcgggtcagTTAATTAATTACCAATGCTTAATCAGTG-3'	To construct *milO* in-frame deletion mutant
14A6-KO-R	5'-ggtggagggcgccgggcaggccggccgatgaccggcgtgTTAATTAATTAGCAGAGCGAGGTATGTA-3'	
14A6-KN-F	5'-tggcccggctgcggacgcttctcgcccgctatgcgtgagTTAATTAATTACCAATGCTTAATCAGTG-3'	To construct *milN* in-frame deletion mutant
14A6-KN-R	5'-attcatggtcactcccatttccgtacggtggggggtcacTTAATTAATTAGCAGAGCGAGGTATGTA-3'	
milO-F	5'-GGCTGATCTCTTCGCGGTGCCTGCCT-3'	To confirm the deletion of *milO* and *milN*
milO-R	5'-CCAACAGGCCCGCTTCCTCATCCTCC-3'	
milN-F	5'-TCCGGCCACGGCATCCACGTCCTGC-3'	
milN-R	5'-GATCAGCGCGGCCACCCGGTCAAGC-3'	
pPM927-F	5'-AGTGCCACCTGACGTCTAAGAAACC-3'	To confirm the overexpression of *milO* and the complementation of *milO* and *milN*
pPM927-R	5'-CGTAGTTCGTGGGCTGGTGGAGTGC-3'	
hrdB-F	5'-AAGCTGACCAGATTCCGGCC-3'	qPCR analysis
hrdB-R	5'-GGTCGCCTTCTTGGCAGTCA-3'	
milA-F	5'-TTCCAAGACGCTTATCTG-3'	
milA-R	5'-GAAGTTGAACACGACGTT-3'	
milB-F	5'-CGGTGTGATGAGCAGCGGT-3'	
milB-R	5'-TCGGCGGGCGACAGGAATT-3'	
milC-F	5'-CGGGAGGCGCAAGAGATACA-3'	
milC-R	5'-GGTGATCCGTCCCTGCGATT-3'	
milD-F	5'-TTCGTCGCCAGTCTCAAC-3'	
milD-R	5'-CGACGTTGCCGAAGAAGT-3'	
milG-F	5'-AACGCCGACTGCTTCAT-3'	
milG-R	5'-CGGTGAACCGGACGTATC-3'	
milI-F	5'-CGGAACGCGACGTCGTGTA-3'	
milI-R	5'-AACTCCTCGGTGCCGTGCA-3'	
milJ-F	5'-TCTGTACACGCCGTTCTCCG-3'	
milJ-R	5'-TTGGCCAGGTGCACGCCTT-3'	

milK-F	5'-GGCTACTACCTCGTCTTCCT-3'	
milK-R	5'-TCGACAGGTTGAGGTACGA-3'	
milL-F	5'-CGTGCTCACCGATCTCAC-3'	
milL-R	5'-GTCGCCACCAACGAGTC-3'	
milM-F	5'-ACACTCTCGCGCACAACCGT-3'	
milM-R	5'-ACACTCTCGCGCACAACCGT-3'	
milP-F	5'-TGCAGGAAGCGGTCGAAG-3'	
milP-R	5'-TCCTCCTCCTGGAGCTCT-3'	
milQ-F	5'-TTGTCGAGCTGGCCGTGT-3'	
milQ-R	5'-CCAGTCGATGAAGCCCACGA-3'	
milAB-F	5'-AGGTCTACCGGCAGTCGCT-3'	RT-PCR to determine the transcription units of MIL BGC
milAB-R	5'-ACGTCCGCGGCCTTGATCT-3'	
milBC-F	5'-CTGGAGAGCCAGGCCAATGTG-3'	
milBC-R	5'-CGACGATCGCGGCCAGCTT-3'	
milCD-F	5'-GTCGACGAGCTGATCGGGAA-3'	
milCD-R	5'-GAGTTGTGGAAGAACGCGCG-3'	
milDE-F	5'-ATGCGATCCAAGGGCTGGTG-3'	
milDE-R	5'-TACACCGCGCAGTTCCCGTT-3'	
milGH-F	5'-GCCGCGTCTTCCTCAACAG-3'	
milGH-R	5'-CGCGTCCACGTGGTCGGTA-3'	
milIJ-F	5'-GCTGCTGGAACTCGACGCT-3'	
milIJ-R	5'-GCGCGGCGTCCATCAGAT-3'	
milLM-F	5'-CTGCACGTACTGGACCCGG-3'	
milLM-R	5'-AGTGTCGGACATGCCATCC-3'	
milKL-F	5'-TTCGCCGCCTTCCCGCTCT-3'	
milKL-R	5'-AGTCAGCCCTGGTGGTCAT-3'	
milOP-F	5'-CGTTCCCTCGCCGCTGTGTT-3'	
milOP-R	5'-TCAAGGAGGTGACCGCCCCGGT-3'	
pSJ8-F	5'-AACCTGTATTTTCAGGGATCCATGTGCCCCCTGACGGAGGC-3'	Expression and purification of MilO
pSJ8-R	5'-GCCAAGGCCTGTACAGAATTCTCAGCCGGAGAGTCCACGG-3'	
milA-PF	5'-CACTGCCCGCCCGTAATAGT-3'	EMSAs and DNase I footprinting assay
milA-PR	5'-FAM-TCCATGGGCCGATGGTCCCT-3'	
milB-PF	5'-CGGGGTTGTTCCTTTCTGGT-3'	
milB-PR	5'-FAM-CCTACTGGAAGCACGTCGT-3'	
milC-PF	5'-GGAGATCCTCCGGTTCTCCGG-3'	
milC-PR	5'-FAM-CGACGATCGCGGCCAGCTT-3'	
milJ-PF	5'-GCTGCTGGAACTCGACGCT-3'	
milJ-PR	5'-FAM-GCGCGGCGTCCATCAGAT-3'	
milL-PF	5'-CTGCACGTACTGGACCCGG-3'	
milL-PR	5'-FAM-AGTGTCGGACATGCCATCC-3'	
milP-PF	5'-AACAACGTGGTCTTGCCGGC-3'	

milP-PR	5’-FAM-GCCGCACCGTGGTGACACTT-3’	
OBS_{WT}-F	5’-TGTCGCCCGGTCGTGTCGCTCGGTGGTGTCCGCCGGTGCCCCGG-3’	EMSAs
OBS_{WT}-R	5’-FAM-CCGGGGCACCGGCGGACACCACCGAGCGACACGACCGGGCGACA-3’	
OBS_{M1}-F	5’-CACTATTTAAT**CG**TGTCGCTCGGTGGTGTCCGCCGGTGCCCCGG-3’	
OBS_{M1}-R	5’-FAM-CCGGGGCACCGGCGGACACCACCGAGCGACACGATTAAATAGTG-3’	
OBS_{M2}-F	5’-TGTCGCCCGGTCGTATTATCTAACGGTGTCCGCCGGTGCCCCGG-3’	
OBS_{M2}-R	5’-FAM-CCGGGGCACCGGCGGACACCGTTAGATAATACGACCGGGCGACA-3’	
OBS_{M3}-F	5’-TGTCGCCCGGTCGTGTCGCTCGGTGGCGCCTATCAACGCCCCGG-3’	
OBS_{M3}-R	5’-FAM-CCGGGGCGTTGATAGGCGCCACCGAGCGACACGACCGGGCGACA-3’	
OBS_{M4}-F	5’-CACTATTTAATCGTATTATCTAACGGTGTCCGCCGGTGCCCCGG-3’	
OBS_{M4}-R	5’-FAM-CCGGGGCACCGGCGGACACCGTTAGATAATACGATTAAATAGTG-3’	
OBS_{M5}-F	5’-TGTCGCCCGGTCGTATTATCTAACGGCGCCTATCAACGCCCCGG-3’	
OBS_{M5}-R	5’-FAM-CCGGGGCGTTGATAGGCGCCGTTAGATAATACGACCGGGCGACA-3’	
OBS_{M6}-F	5’-CACTATTTAATCGTGTCGCTCGGTGGCGCCTATCAACGCCCCGG-3’	
OBS_{M6}-R	5’-FAM-CCGGGGCGTTGATAGGCGCCACCGAGCGACACGATTAAATAGTG-3’	
OBS_{M7}-F	5’-CACTATTTAATCGTATTATCTAACGGCGCCTATCAACATCCCGG-3’	
OBS_{M7}-R	5’-FAM-CCGGGATGTTGATAGGCGCCGTTAGATAATACGATTAAATAGTG-3’	

Table S4 The reference proteins for sequence alignment and phylogenetic analysis

Protein name	Accession	Source
MilO	AFD20755.1	*Streptomyces rimofaciens*
-	WP_190160668.1	*Streptomyces litmocidini*
-	WP_052706645.1	*Streptomyces rubellomurinus*
-	WP_178379169.1	*Streptomyces sp.* NBRC 110465
Cgc1	AKZ59702.1	*Streptomyces ambofaciens*
ChiR	BAA88407.1	*Streptomyces thermoviolaceus*
CepR	EFG08031.1	*Streptomyces clavuligerus*

EcrE2	GAA2690875.1	*Streptomyces violaceolatus*
PapR6	WP_005321842.1	*Streptomyces pristinaespiralis*
NcnR	WP_133023426.1	*Streptomyces arenae*
RedZ	CAA69209.1	*Streptomyces coelicolor*A3(2)
NysRIII	AAF71780.1	*Streptomyces noursei* ATCC 11455
NysRI	AAF71778.1	*Streptomyces noursei* ATCC 11455
RapH	AAC38065.1	*Streptomyces hygroscopicus*
SlnR	AEZ53964.1	*Streptomyces albus*
PikD	AAC68887.1	*Streptomyces venezuelae*
SsaA	KOG30995.1	*Streptomyces viridochromogenes*
PacA	ADN26237.1	*Streptomyces coeruleorubidus*
SrosN15	USC48768.1	*Streptomyces filamentosus*
Mur33	ADZ45345.1	*Streptomyces sp.* NRRL 30471
FscRI	ALM43429.1	*Streptomyces sp.* FR-008
TtmRIV	AFQ68276.1	*Streptomyces hygrospinosus*
AURJ3M	ACD75765.1	*Streptomyces aureofuscus*
FiIF	WP_191875315.1	*Streptomyces filipinensis*

Table S5 ^{1}H NMR and ^{13}C NMR data for compound **1** in CD_3OD (700 MHz)

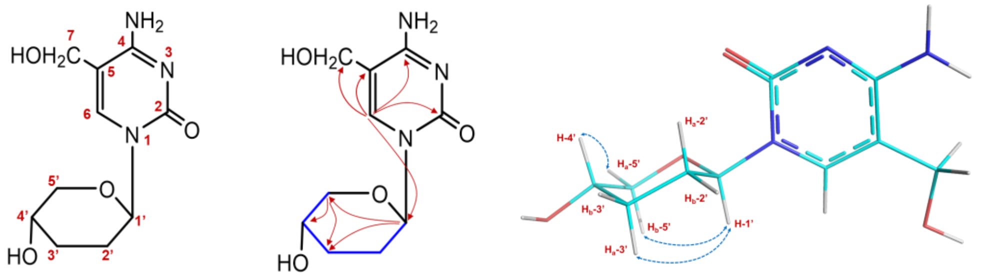

Chemical structure of compound **1** and ^{1}H-^{1}H COSY (▬), key HMBC correlations (→), selected NOESY correlations (◄- - - - -►)

No.	^{13}C	^{1}H (muit. *J*, Hz)
2	160.2, C	
4	164.7, C	
5	107.6, C	
6	143.8, CH	8.01 (s)
7	57.9, CH_2	4.47 (s)
1’	84.0, CH	5.59 (dd, 10.8, 2.5)
2’	30.1, CH_2	1.77 (tdd. 13.3, 10.8, 4.0, H_a)
		2.03 (m, H_b)
3’	32.1, CH_2	1.62 (tdd. 13.1, 10.9, 4.0, H_a)
		2.19 (m, H_b)
4’	65.3, CH	3.74 (m)
5’	73.6, CH_2	3.37 (m, H_b)
		4.09 (ddd, 11.0, 5.1, 2.4, H_a)

Table S6 ^{1}H NMR and ^{13}C NMR data for compound **2** in CD_3OD (700 MHz)

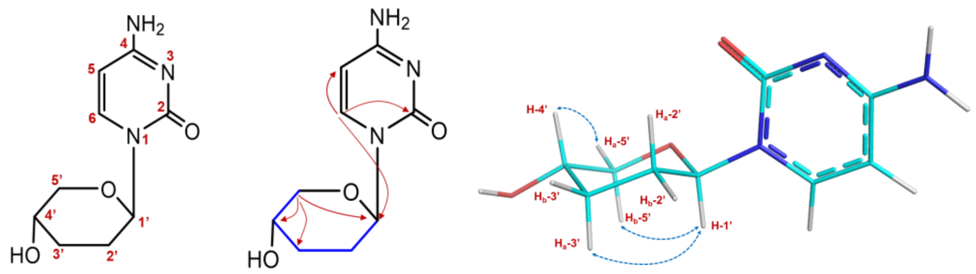

Chemical structure of compound **2** and ^{1}H-^{1}H COSY (—), key HMBC correlations (→), selected NOESY correlations (↔)

No.	^{13}C	^{1}H (muit. *J*, Hz)
2	161.3, C	
4	165.0, C	
5	95.0, CH	6.11 (d, 7.8)
6	146.3, CH	8.03 (d, 7.9)
1’	84.1, CH	5.59 (dd, 10.8, 2.3)
2’	30.2, CH_2	1.75 (tdd, 13.3, 10.8, 4.0, H_a)
		2.03 (dq, 13.1, 3.3, H_b)
3’	32.2, CH_2	1.62 (tdd. 13.0, 10.9, 3.9, H_a)
		2.19 (dt, 12.6, 3.6, H_b)
4’	65.4, CH	3.73 (tq, 10.8, 6.1, 5.5)
5’	73.7, CH_2	3.37 (t, 10.7, H_b)
		4.09 (ddd, 11.0, 5.1, 2.5, H_a)

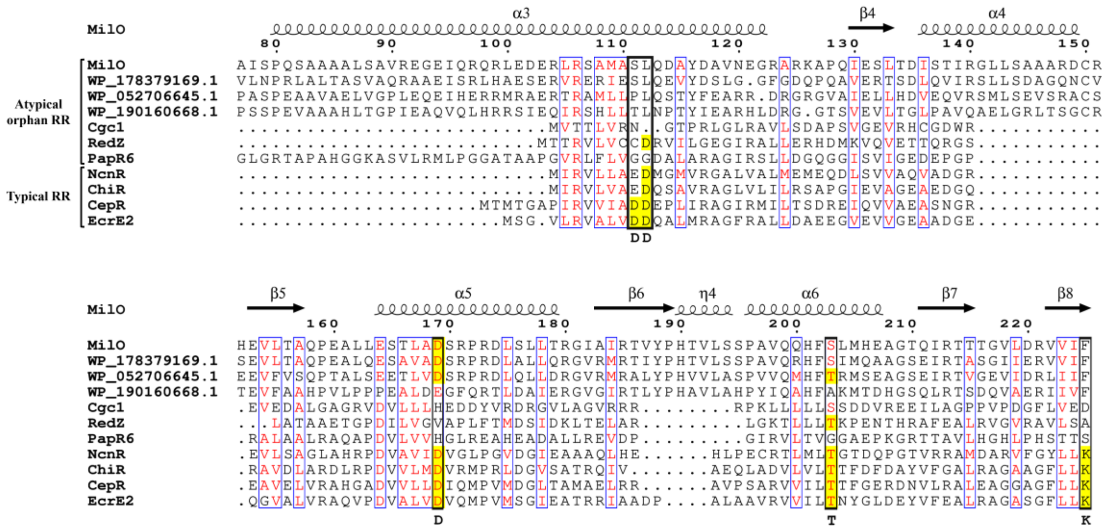

Figure S1 Sequence alignment of putative MilO REC domain and its homologs with other REC-LuxR. The sites of the most conserved phosphorylation residues in typical REC domains were framed in black, and the most conserved amino acids (DD, D, T, and K) were highlighted in yellow.

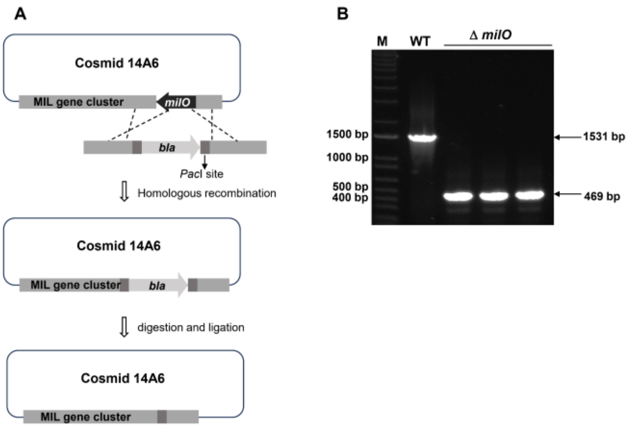

Figure S2 In-frame deletion of *milO* in cosmid 14A6. (A) Schematic diagram of the deletion of *milO* by PCR-targeting. (B) PCR verification of the mutant strain Δ*milO*. Lane M, 1kb DNA ladder. Lane WT, cosmid 14A6 was analyzed as control. Δ*milO*, 14A6Δ*milO*.

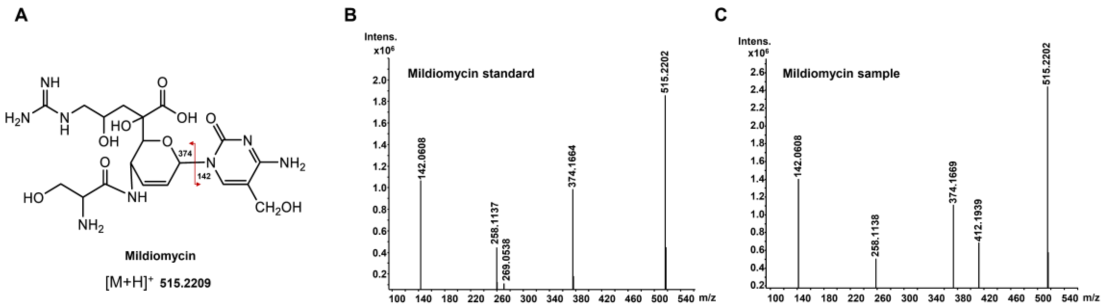

Figure S3 HPLC-Q-TOF-MS analysis of mildiomycin (positive model). (A) The structure of mildiomycin. (B) HPLC-Q-TOF-MS analysis of the mildiomycin standard. (C) HPLC-Q-TOF-MS

analysis of the mildiomycin sample collected from the extract of Δ*milO*::*milO* (*S. avermitilis*::14A6ΔmilO::pPM927-*kasO*p*-*milO*) fermentation. The molecular ion peaks $[M+H]^+$ were both observed at m/z =515.2202 for the standard and the sample.

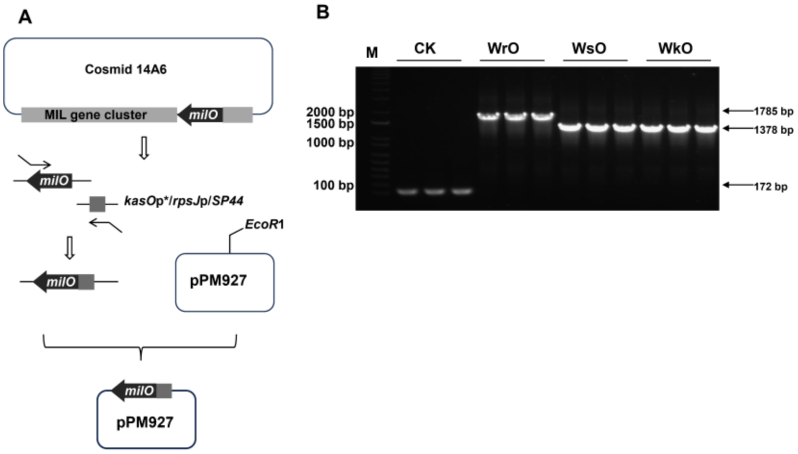

Figure S4 Construction of recombinant plasmids and confirmation of strains for over-expression of *milO* with different promoters. (A) Schematic diagram of the construction of pPM927-1, pPM927-2 and pPM927-3 by overlap PCR. (B) PCR verification of the strains WrO, WsO and WkO. Lane M, 1kb DNA ladder. Lane CK, WT::pPM927. Lane WrO, WT::pPM927-3. Lane WsO, WT::pPM927-2. Lane WkO, WT::pPM927-1. WT denotes *S. avermitilis*::14A6.

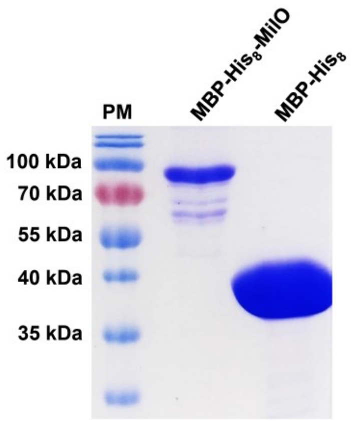

Figure S5 SDS-PAGE analysis of purified MBP-His_8-MilO and MBP-His_8.

A

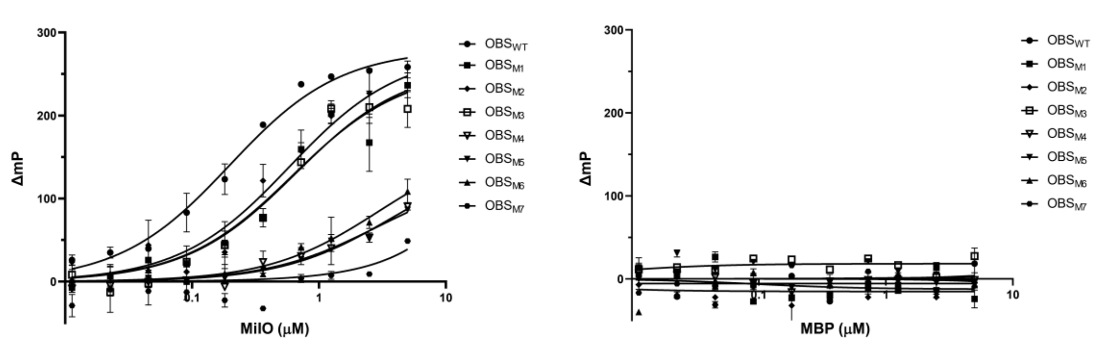

B

	Probe	OBS_{WT}	OBS_{M1}	OBS_{M2}	OBS_{M3}	OBS_{M4}	OBS_{M5}	OBS_{M6}	OBS_{M7}
K_D (μM)	MilO	0.1976	0.6234	0.5857	0.6551	3.766	2.859	2.951	ND
	MBP	ND	ND	ND	ND	ND	ND	ND	ND

Figure S6 Fluorescence polarization assays of the MilO and MBP with OBS_{WT} and its variants. (A) Binding curves of MilO and MBP to probes. Error bars were calculated by measuring the standard deviations of the data from three replicates of each sample. (B) The dissociation constant (K_D) of the proteins, MilO and MBP, binding to probes. "ND" means the K_D value is too large to be determined and not credible.

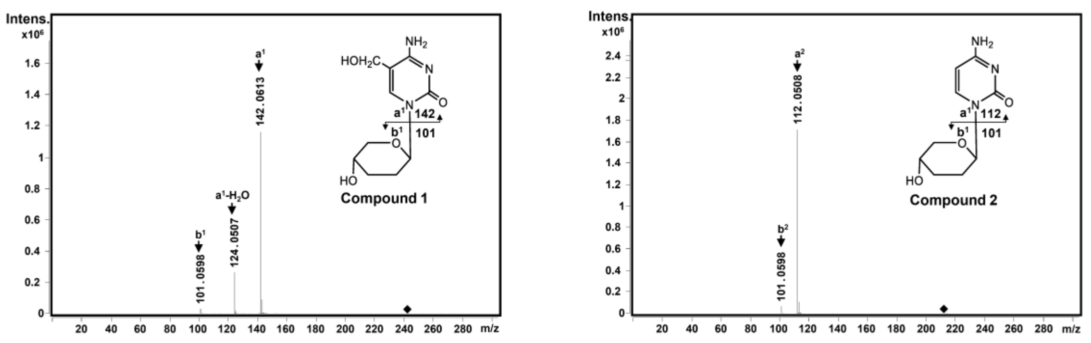

Figure S7 HPLC-Q-TOF-MS/MS analysis of compound **1** (A) and compound **2** (B). The fragment a^1 of **1**: Cal. 142.0611, Obs. 142.0613, 1.41 ppm Error, b^1: Cal. 101.0597, Obs. 101.0598, 0.99 ppm Error, a^1-H_2O: Cal. 124.0505, Obs. 124.0507, 1.61ppm Error; the fragment a^2 of **2**: Cal. 112.0505, Obs. 112.0508, 2.67 ppm Error, b^2: Cal. 101.0597, Obs. 101.0598, 0.99 ppm Error.

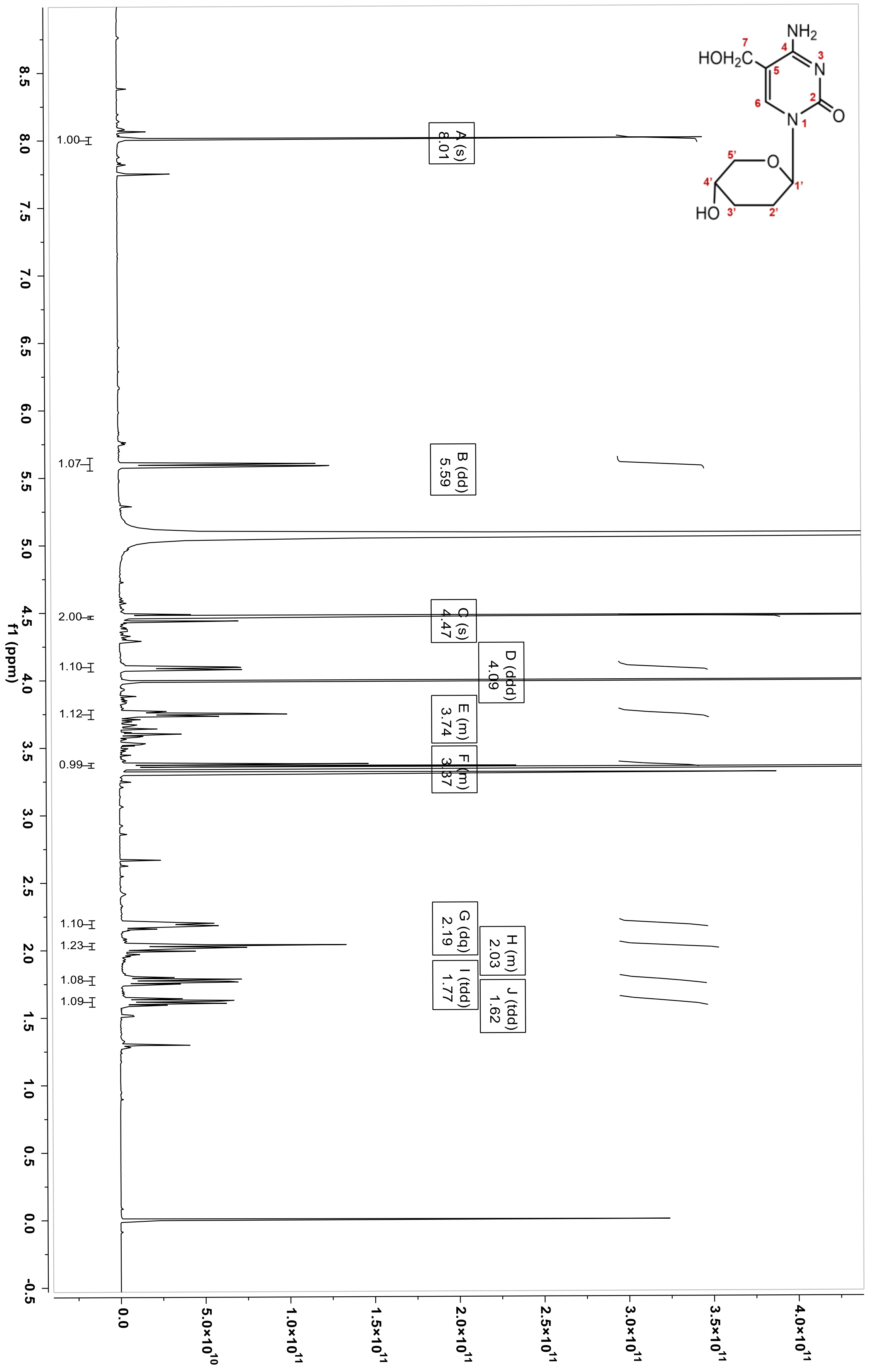

Figure S8 ^{1}H NMR data for compound **1** in CD_3OD

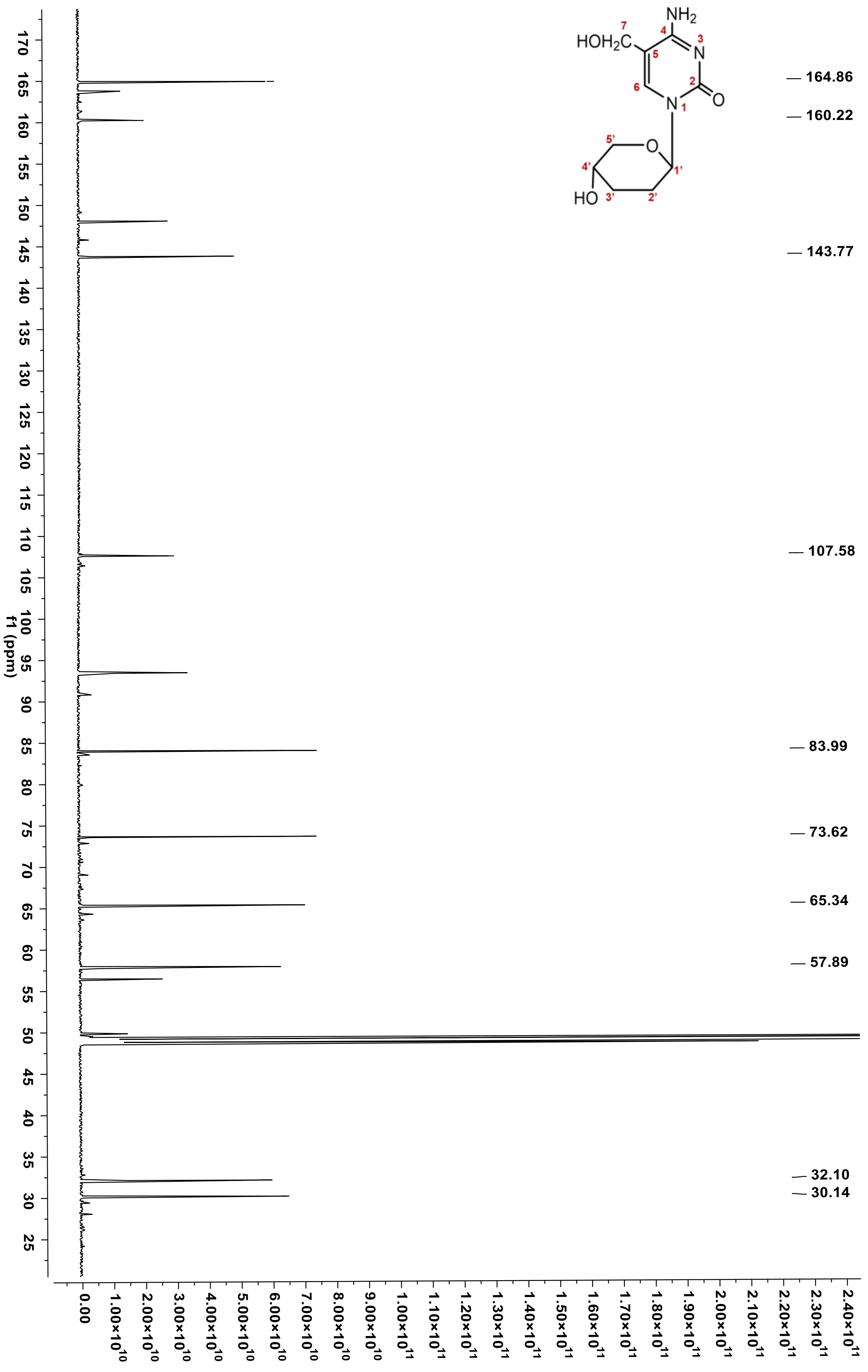

Figure S9 ^{13}C NMR data for compound **1** in CD_3OD

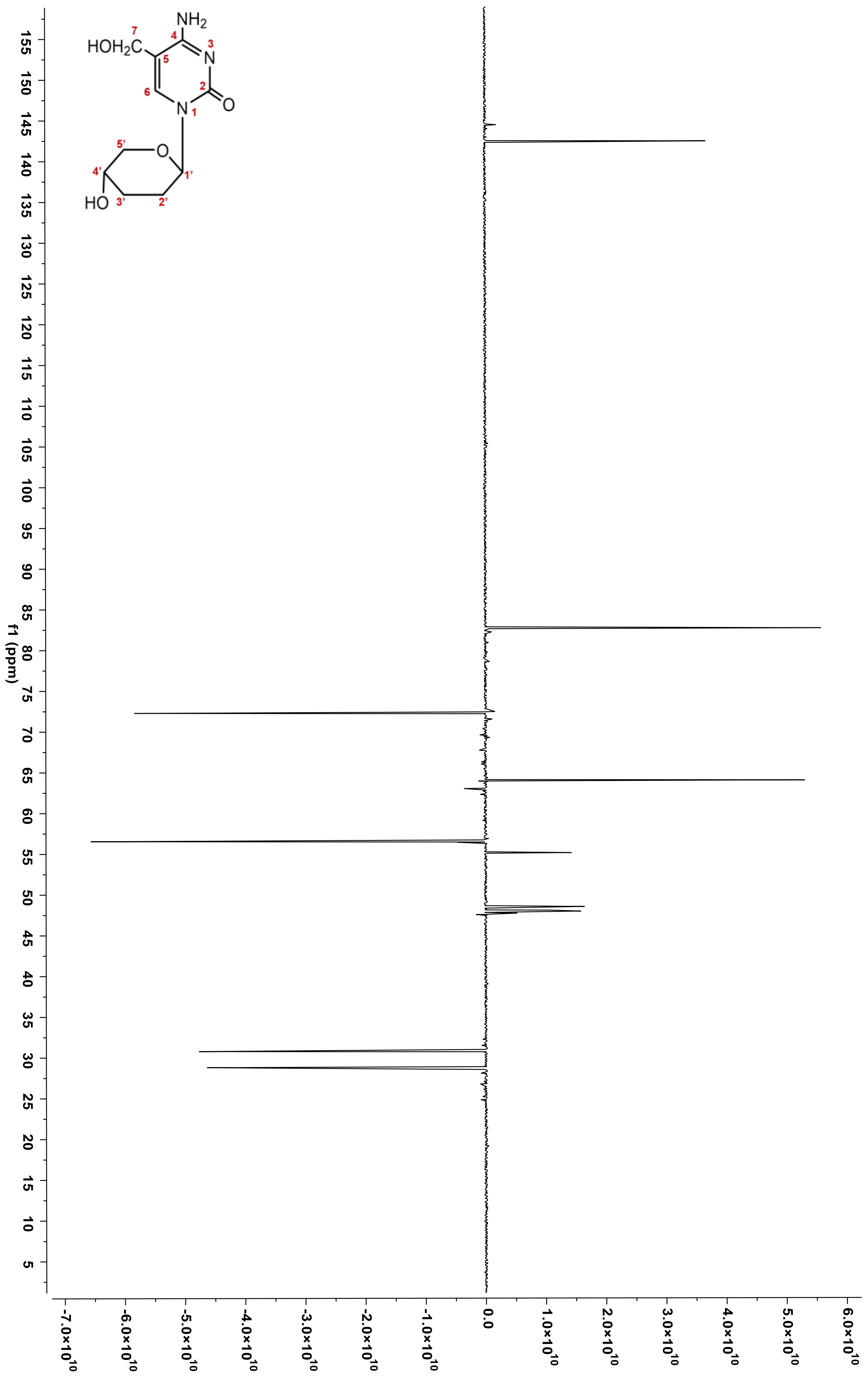

Figure S10 DEPT135 spectrum of compound **1** in CD_3OD

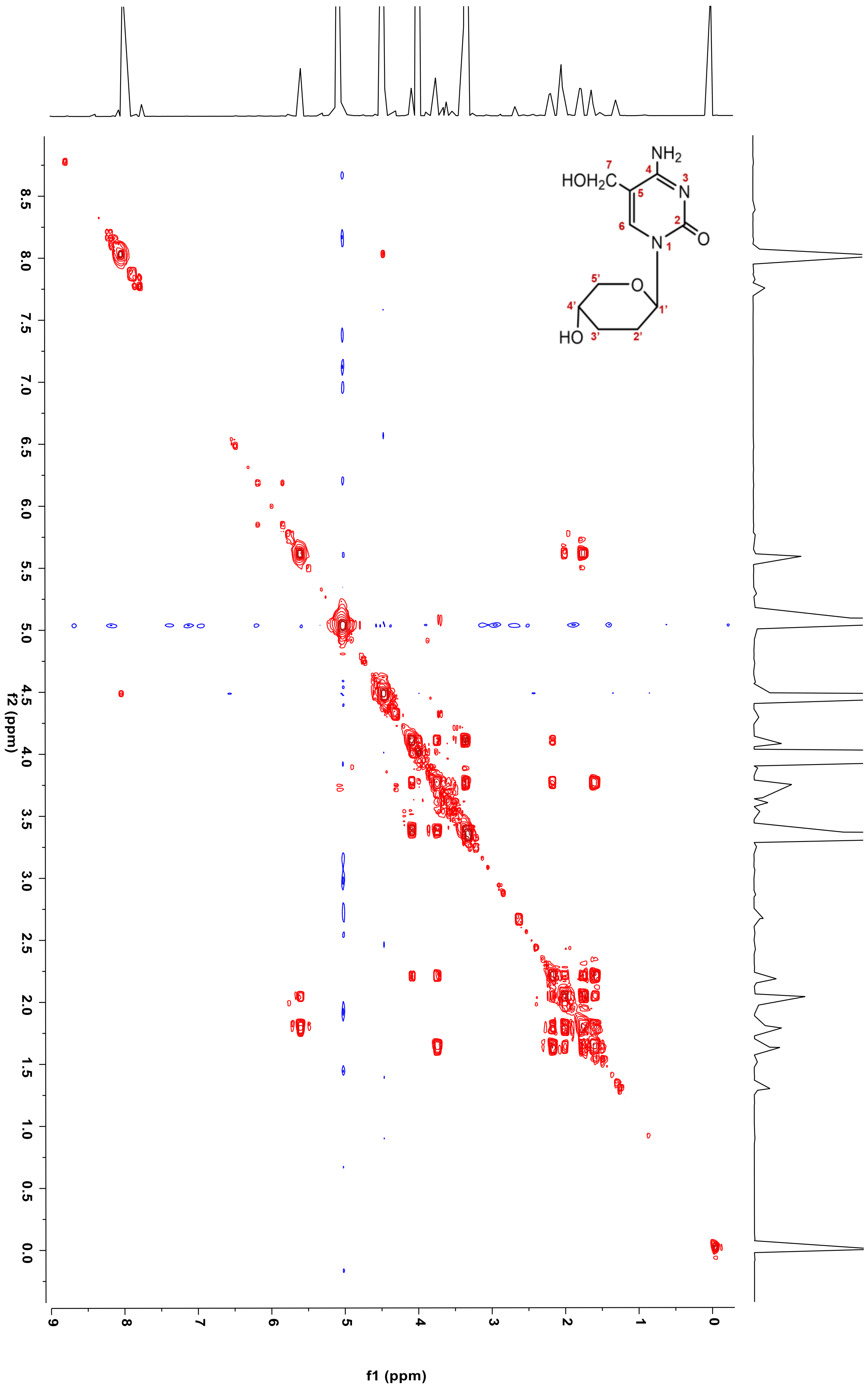

Figure S11 COSY data of compound **1** in CD_3OD

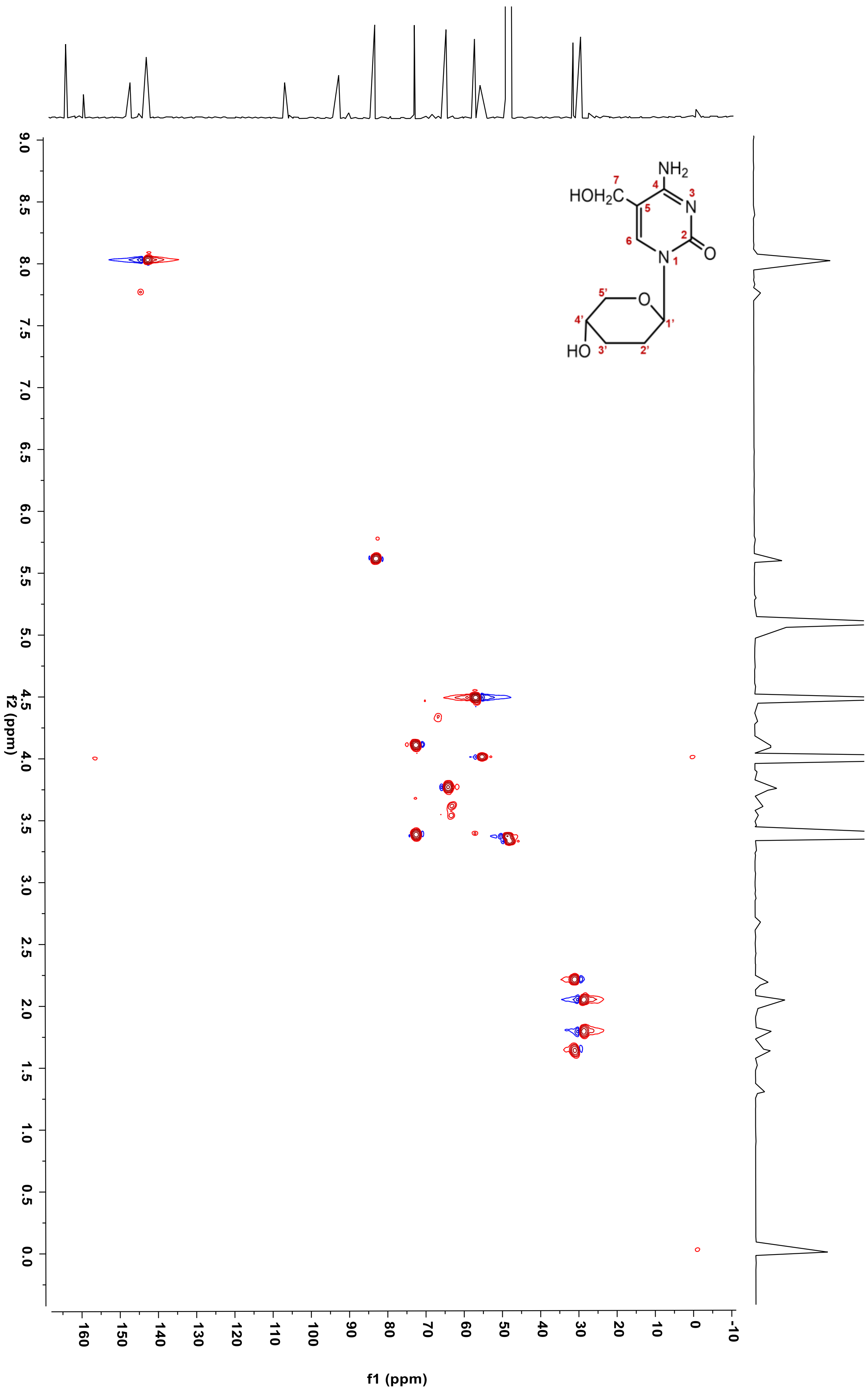

Figure S12 HSQC spectrum of compound **1** in CD_3OD

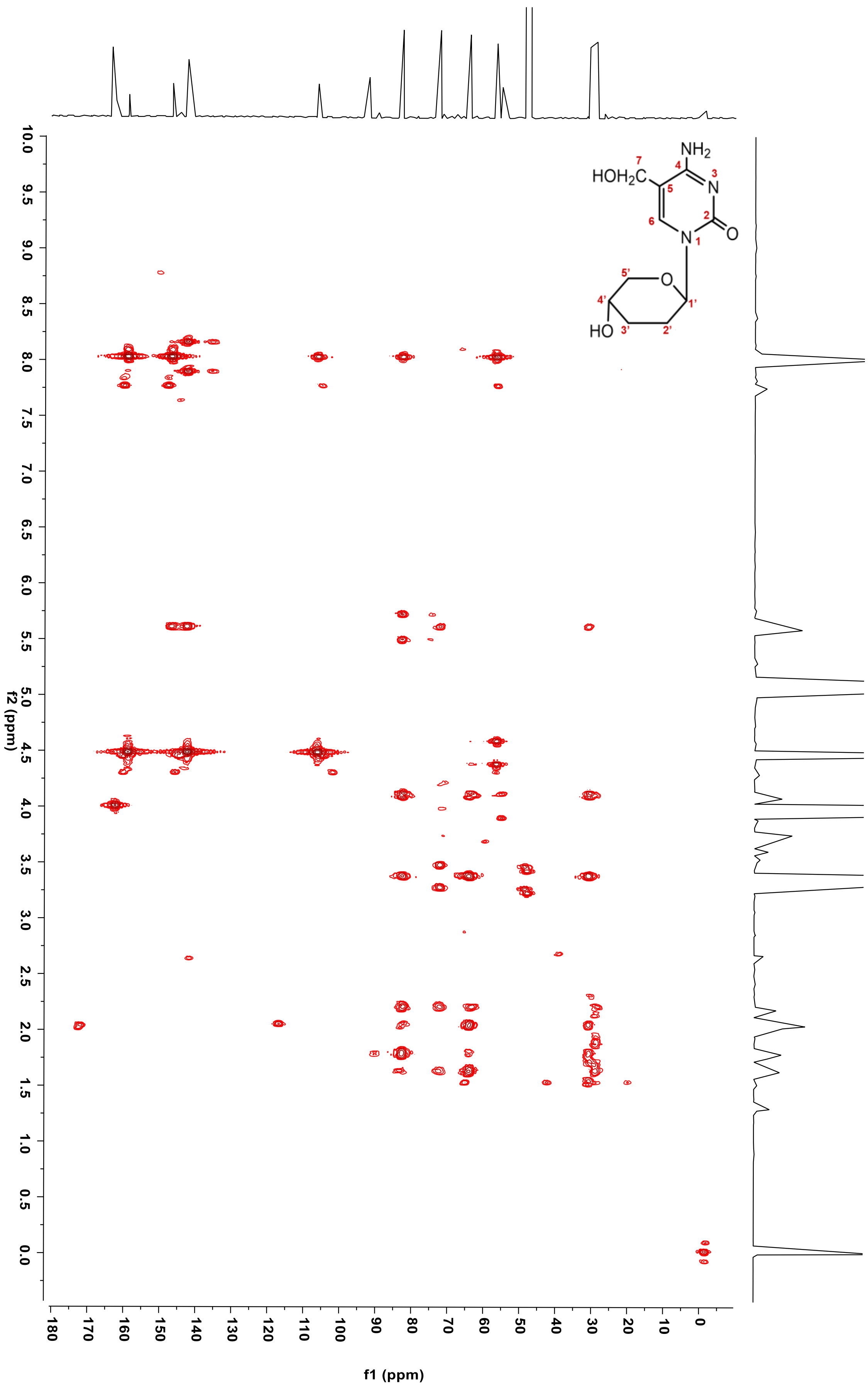

Figure S13 HMBC spectrum of compound **1** in CD_3OD

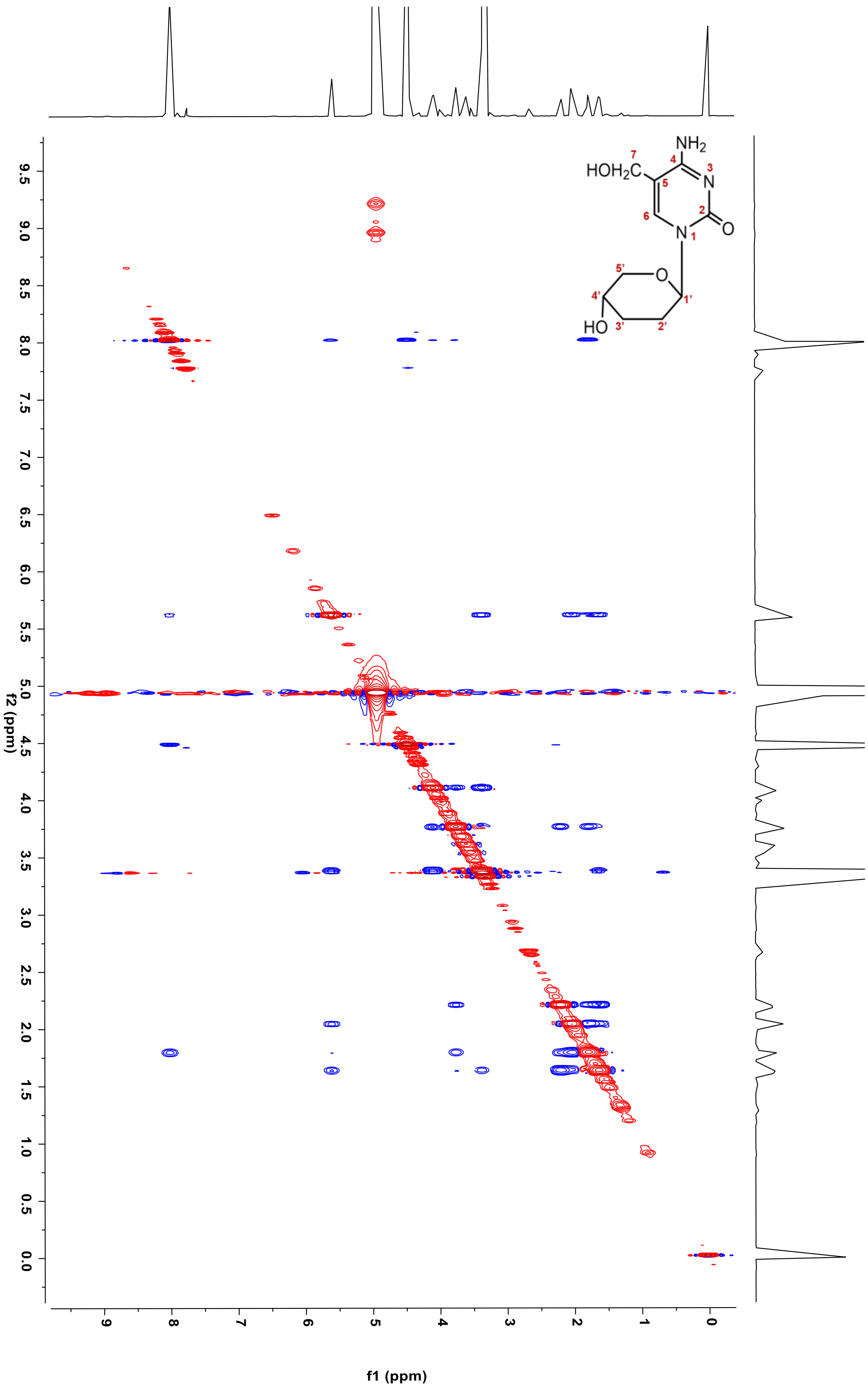

Figure S14 NOESY spectrum of compound **1** in CD_3OD

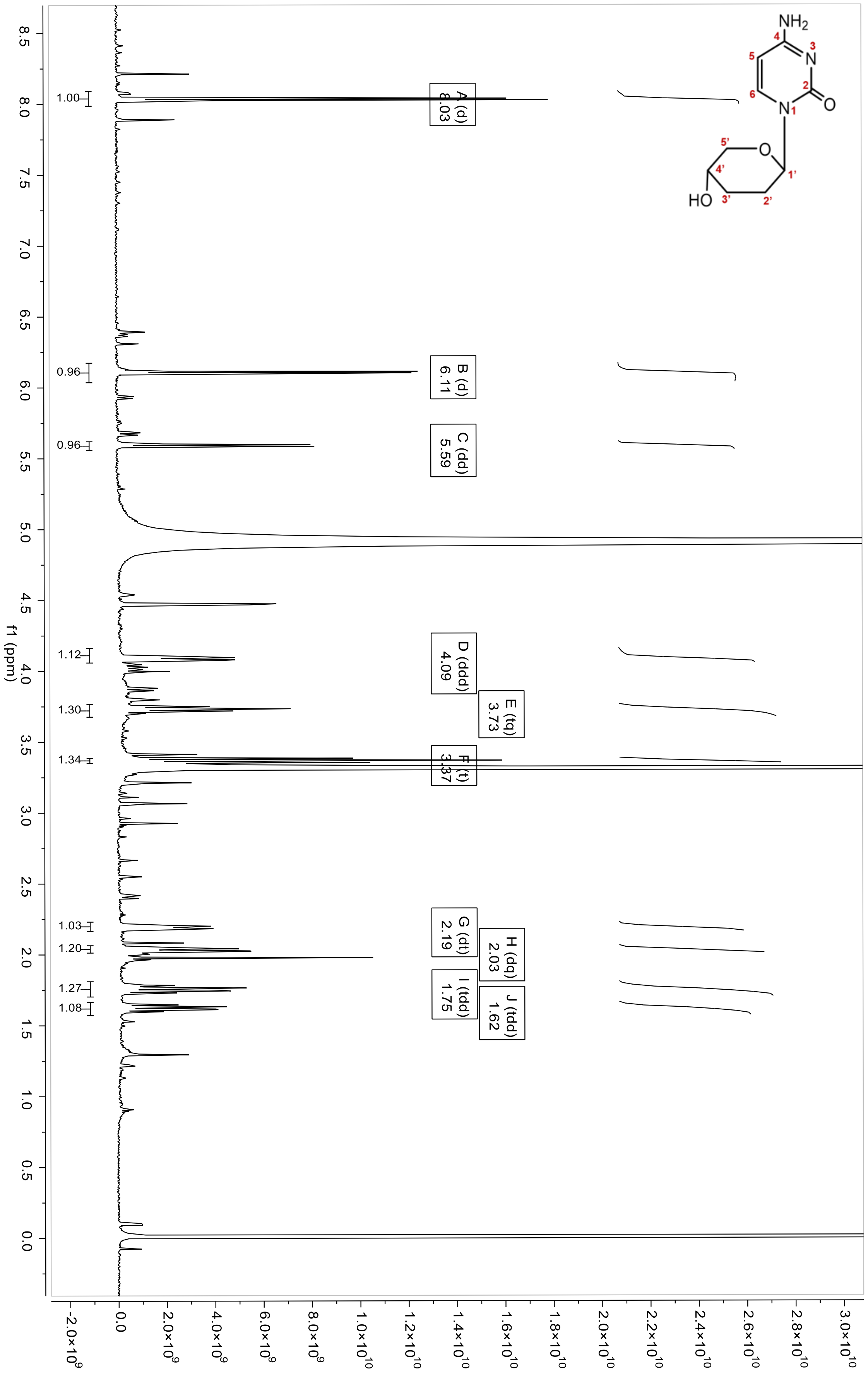

Figure S15 ^{1}H NMR data for compound **2** in CD_3OD

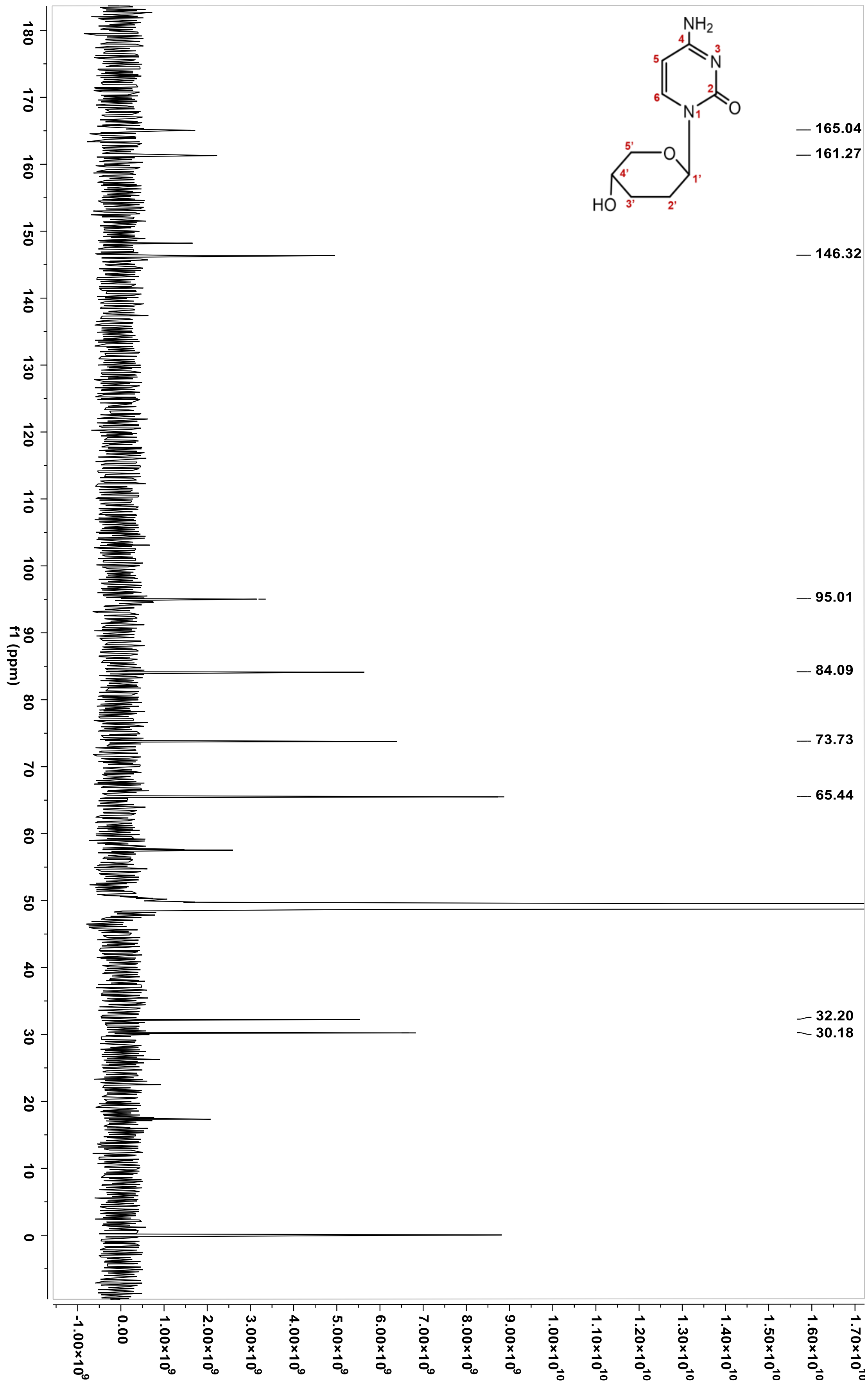

Figure S16 ^{13}C NMR data for compound **2** in CD_3OD

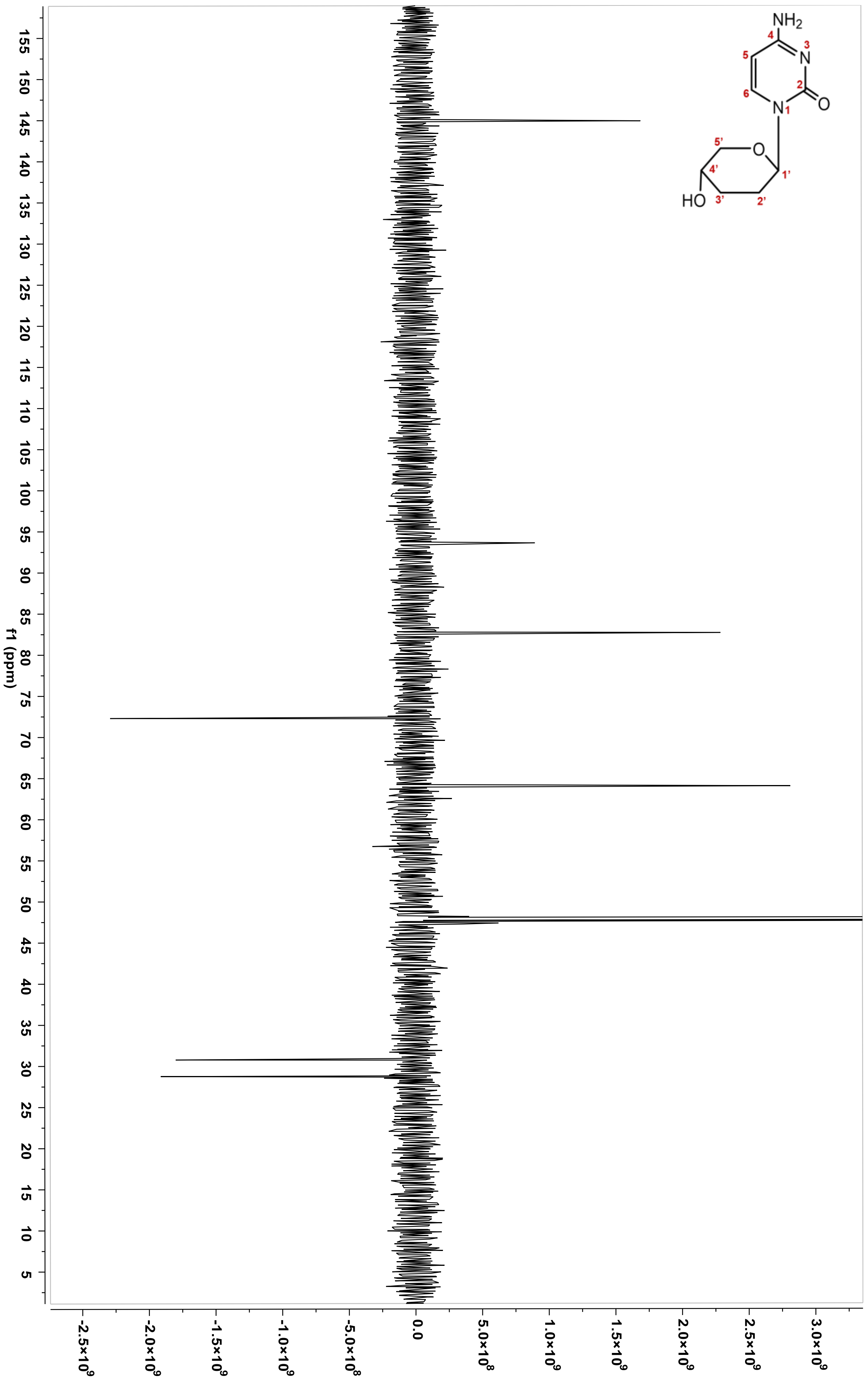

Figure S17 DEPT135 spectrum of compound **2** in CD_3OD

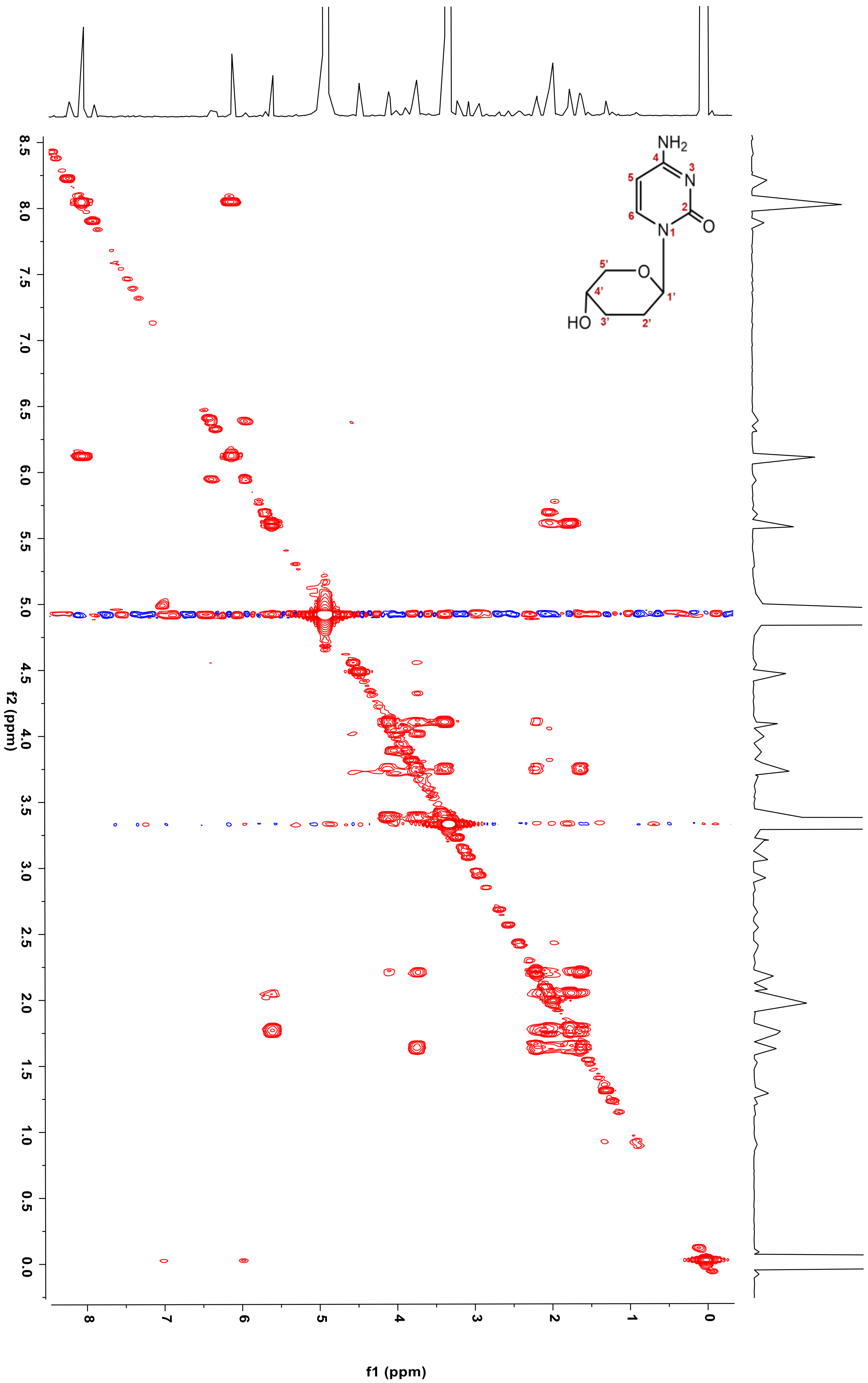

Figure S18 COSY data of compound **1** in CD_3OD

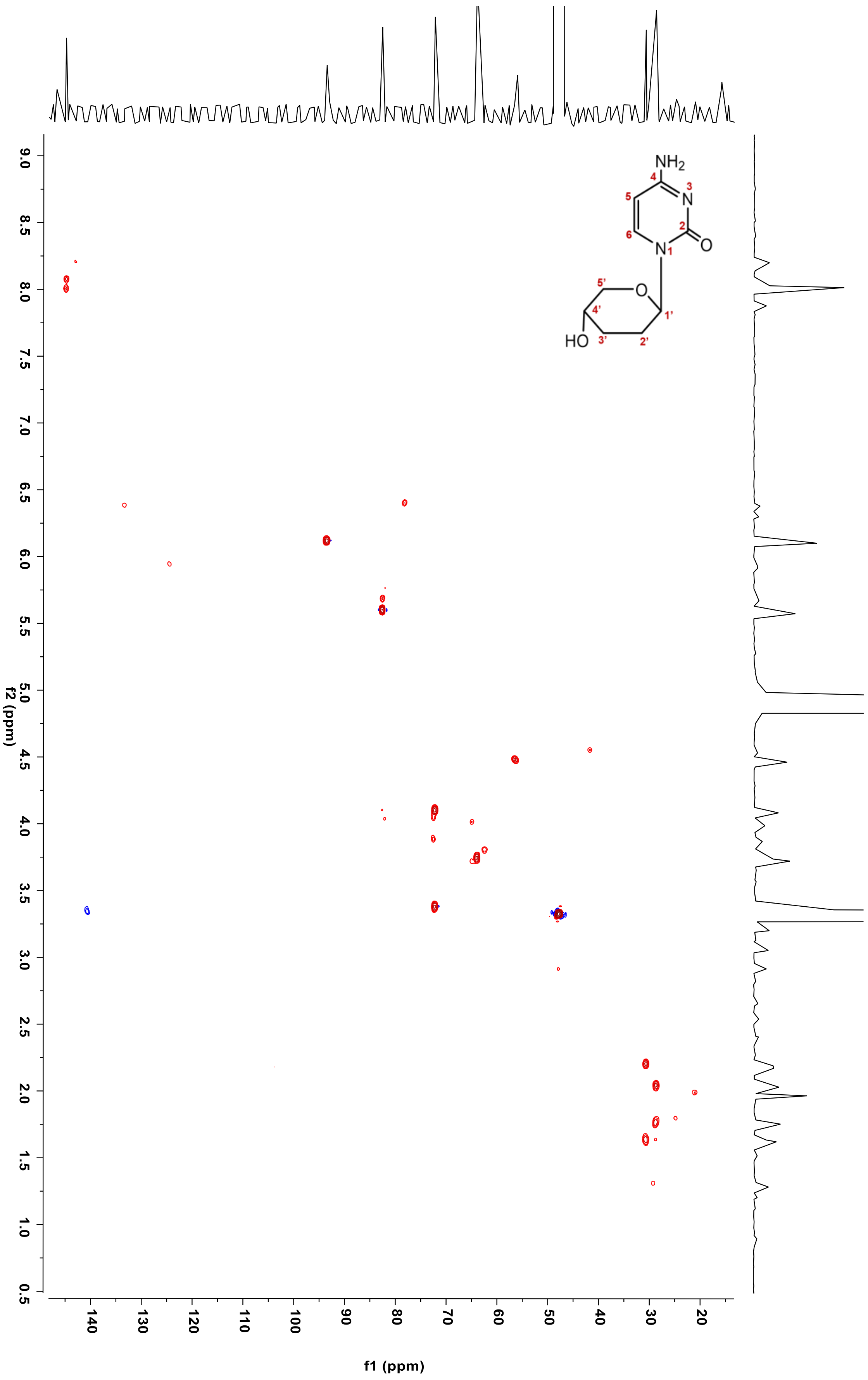

Figure S19 HSQC spectrum of compound **1** in CD_3OD

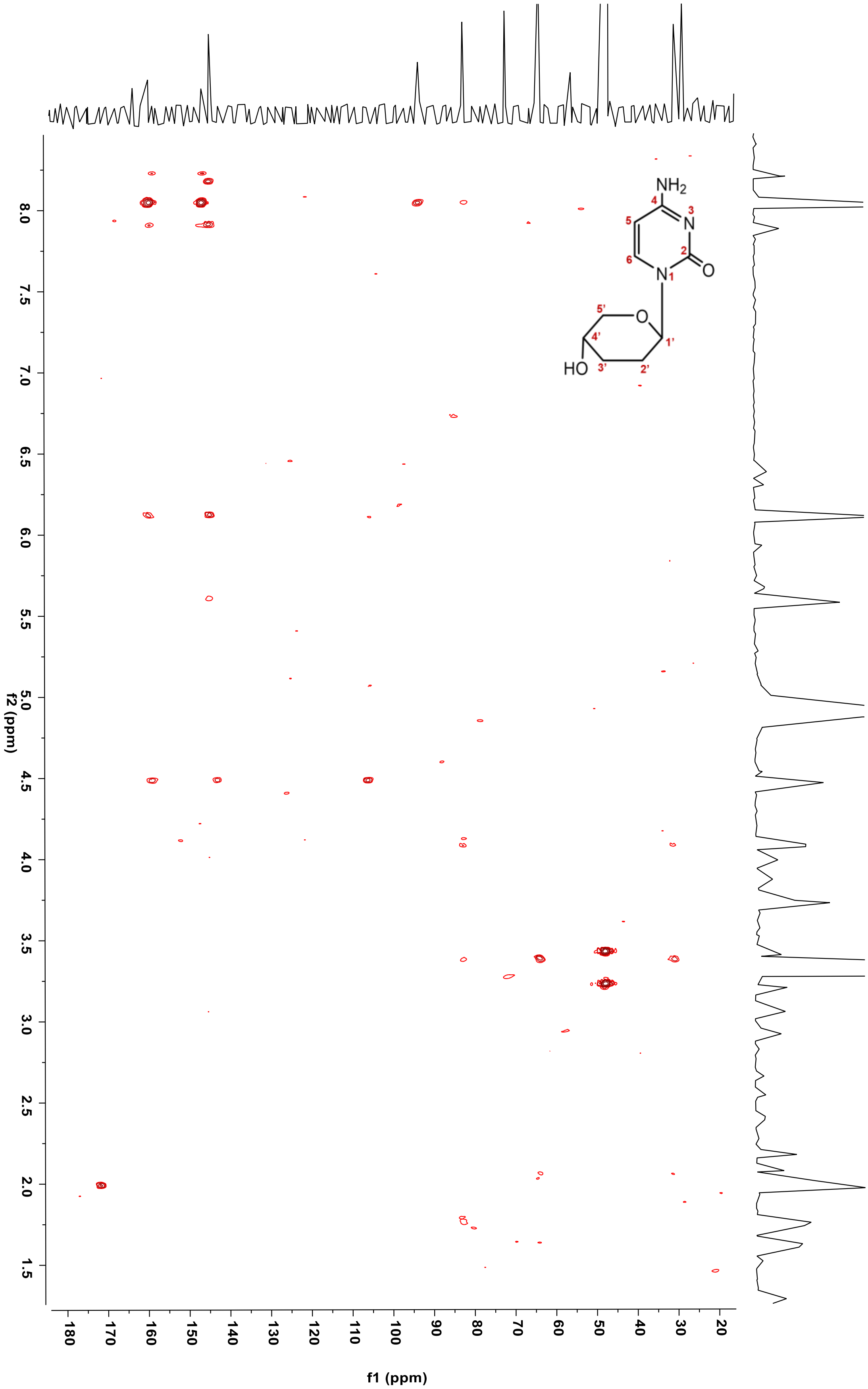

Figure S20 HMBC spectrum of compound **1** in CD_3OD

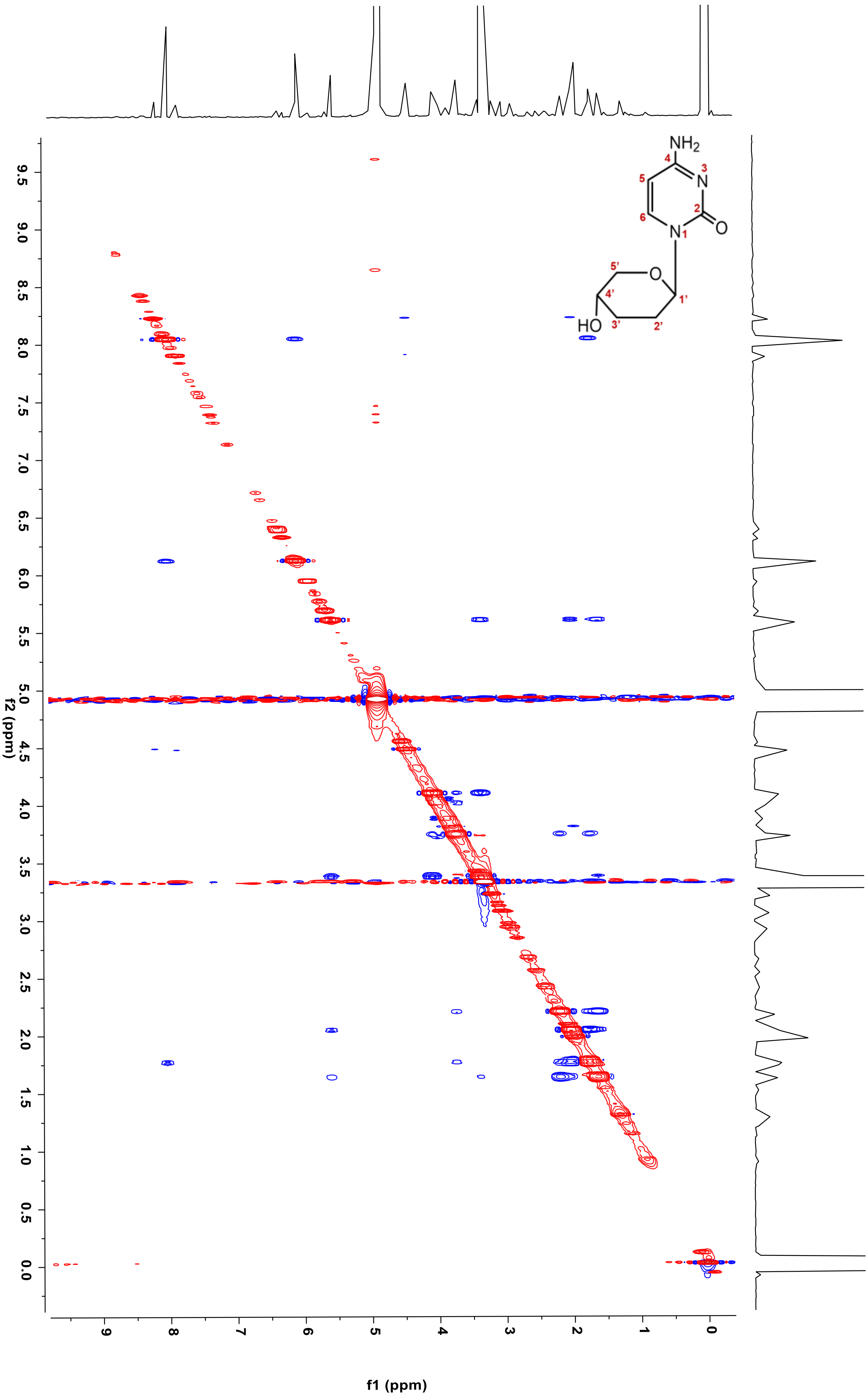

Figure S21 NOESY spectrum of compound **1** in CD_3OD

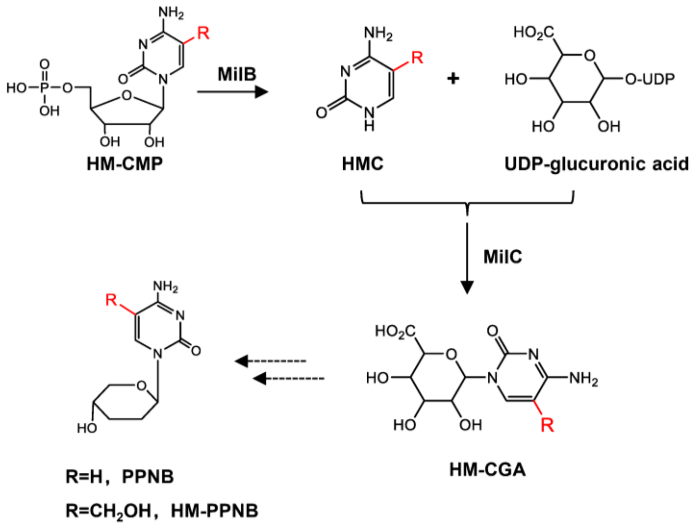

Figure S22 The proposed synthesis of PPNB and HM-PPNB in the biosynthesis pathway of mildiomycin.

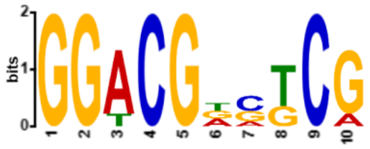

milAp tgtcgcccGGTCGTGTCGctcggtgg
milBp gcacgcccGGACGACGCGaccccacc
milCp ggcggactGGACGGCTCGggcggacg
milJp ggcggactGGACGGCTCGggcggacg
milMp ccccggagGGACGTCTCAagggacgt
milQp cgggggctGGACGAGGCGgtggcctc

Figure S23 Comparison of the *milO* binding site in the MilA promoter with other *mil* operons. The conserved sequences were underlined by horizontal line.

References

1. Li L, Xu Z, Xu X, Wu J, Zhang Y, He X, Zabriskie TM, Deng Z. 2008. The mildiomycin biosynthesis: initial steps for sequential generation of 5-hydroxymethylcytidine 5'-monophosphate and 5-hydroxymethylcytosine in *Streptoverticillium rimofaciens* ZJU5119. Chembiochem : a European Journal of Chemical Biology 9:1286-1294.
2. Wu J, Li L, Deng Z, Zabriskie TM, He X. 2012. Analysis of the mildiomycin biosynthesis gene cluster in *Streptoverticillum remofaciens* ZJU5119 and characterization of MilC, a hydroxymethyl cytosyl-glucuronic acid synthase. Chembiochem : a European Journal of Chemical Biology 13:1613-1621.
3. Hoffarth ER, Caddell Haatveit K, Kuatsjah E, MacNeil GA, Saroya S, Walsby CJ, Eltis LD, Houk KN, Garcia-Borràs M, Ryan KS. 2021. A shared mechanistic pathway for pyridoxal phosphate-dependent arginine oxidases. Proceedings of the National Academy of Sciences of the United States of America 118.
4. Kieser T, Bibb MJ, Chater KF, Butter M, Hopwood D, Bittner ML, Buttner MJ. Practical *Streptomyces* Genetics: A Laboratory Manual, p. *In* (ed),
5. Gust B, Challis GL, Fowler K, Kieser T, Chater KF. 2003. PCR-targeted *Streptomyces* gene replacement identifies a protein domain needed for biosynthesis of the sesquiterpene soil odor geosmin. Proceedings of the National Academy of Sciences of the United States of America 100:1541-1546.
6. Smokvina T, Mazodier P, Boccard F, Thompson CJ, Guérineau M. 1990. Construction of a series of pSAM2-based integrative vectors for use in actinomycetes. Gene 94:53-9.
